# The Cost Effectiveness of Pandemic Influenza Interventions: A Pandemic Severity Based Analysis

**DOI:** 10.1371/journal.pone.0061504

**Published:** 2013-04-09

**Authors:** George J. Milne, Nilimesh Halder, Joel K. Kelso

**Affiliations:** Simulation and Modelling Research Unit, University of Western Australia, Perth, Australia; The University of Hong Kong, Hong Kong

## Abstract

**Background:**

The impact of a newly emerged influenza pandemic will depend on its transmissibility and severity. Understanding how these pandemic features impact on the effectiveness and cost effectiveness of alternative intervention strategies is important for pandemic planning.

**Methods:**

A cost effectiveness analysis of a comprehensive range of social distancing and antiviral drug strategies intended to mitigate a future pandemic was conducted using a simulation model of a community of ∼30,000 in Australia. Six pandemic severity categories were defined based on case fatality ratio (CFR), using data from the 2009/2010 pandemic to relate hospitalisation rates to CFR.

**Results:**

Intervention strategies combining school closure with antiviral treatment and prophylaxis are the most cost effective strategies in terms of cost per life year saved (LYS) for all severity categories. The cost component in the cost per LYS ratio varies depending on pandemic severity: for a severe pandemic (CFR of 2.5%) the cost is ∼$9 k per LYS; for a low severity pandemic (CFR of 0.1%) this strategy costs ∼$58 k per LYS; for a pandemic with very low severity similar to the 2009 pandemic (CFR of 0.03%) the cost is ∼$155 per LYS. With high severity pandemics (CFR >0.75%) the most effective attack rate reduction strategies are also the most cost effective. During low severity pandemics costs are dominated by productivity losses due to illness and social distancing interventions, while for high severity pandemics costs are dominated by hospitalisation costs and productivity losses due to death.

**Conclusions:**

The most cost effective strategies for mitigating an influenza pandemic involve combining sustained social distancing with the use of antiviral agents. For low severity pandemics the most cost effective strategies involve antiviral treatment, prophylaxis and short durations of school closure; while these are cost effective they are less effective than other strategies in reducing the infection rate.

## Introduction

There is continuing concern that a highly pathogenic H5N1 avian influenza strain may become transmissible between humans. This scenario is highlighted by the large reservoir of H5N1 in poultry in South-East Asia [1], and by recent experimental results which have shown that the H5N1 virus may be genetically modified to become readily transmissible between ferrets, a commonly used animal model for human influenza transmission studies [2]–[4].

The severity of a particular influenza strain directly impacts on the cost of any pandemic; increased severity increases health care costs and escalates productivity losses due to *a*) absenteeism arising from increased illness and *b*) increased mortality rates.

For a future pandemic which is highly pathogenic, a reduction in the attack rate is crucial as this directly reduces the number of lives lost. While a number of intervention strategies may be equally effective in reducing the illness attack rate, the ability to determine the total cost of each intervention also permits the cost effectiveness of a given intervention strategy to be determined. Comparison of interventions is then possible from both an effectiveness and cost effectiveness standpoint, with these analyses being conducted for each severity class, as presented here.

The need for an unambiguous, extended definition of severity has been noted in the World Health Organization report on the handling of the 2009 pandemic [5], which highlights the impact pandemic severity has on health care provision and associated costs. In the absence of such definitions, an extended severity metric is presented. This utilises severity categories 1 through 5 as proposed by the CDC, based on the case fatality ratio (CFR) [6], adds an additional category 0 to reflect the “mild” severity of the 2009 pandemic, and extends the severity definition to include hospitalisation and intensive care unit (ICU) rates, using data collected in Australia during the 2009/2010 pandemic. These data have been used to generate a more extensive notion of severity, by linking case fatality rates with hospitalisation rates.

Results from this study indicate which intervention strategies are most cost effective when considering the total costs of a pandemic, including productivity losses to the economy due to absenteeism and death. The role which pandemic severity has on the cost effectiveness of a range of potential intervention strategies is determined, and for highly pathogenic influenza strains inducing significant morbidity and mortality, such as occurred during the 1918/1919 pandemic [7], [8], this study determines which intervention strategies are the most effective and most cost effective.

## Methods

A detailed, individual-based simulation model of a real community in the south-west of Western Australia, the town of Albany with a population of approximately 30,000 was used to simulate the dynamics of an influenza pandemic. Comparing simulations with and without interventions in place allowed a determination to be made on the effect which a range of interventions have on reducing the illness attack rate and on the health of each individual in the modelled community. Data produced by the simulation model were used to determine health outcomes involving hospitalisation, ICU treatment, and death. In turn, these healthcare outcomes, together with productivity losses due to removal from the workforce, were used to estimate the cost and cost effectiveness of interventions; cost effectiveness being presented as net cost per Life Year Saved (LYS).

### Simulation Model

The individual-based simulation model was developed using census, state, and local government data to construct a human contact network involving households, schools, childcare centres, workplaces, and a regional hospital. Census data was used to populate each household in our modelled community with the exact number of uniquely identified individuals, with ages in one of seven age groups (0–5, 6–12, 13–17, 18–24, 25–44, 45–64, 65+). These ages were used to allocate specific children to appropriate schools and classes, and adults to workplaces, using data on workplaces and schools in the community. This model was previously developed to determine the effectiveness of social distancing and vaccination measures for a possible future H5N1 pandemic [9]–[11], and was subsequently used to examine antiviral and school closure interventions which were employed in the H1N1 2009 influenza pandemic [12]–[14], and to determine the cost effectiveness of such interventions in a low severity 2009 pandemic setting [15]. Age-based susceptibility of individuals to infection was calibrated to reproduce age-specific infection rates observed in the 2009 pandemic using data from [16]. An alternative age-based susceptibility profile, where all age groups were equally susceptible, was used in a sensitivity analysis. It was assumed that 32% of adult infections and 20% of child infections [17] (an average of 30% across all ages) were asymptomatic; an alternative asymptomatic rate of 60% [18] was used in a sensitivity analysis.

The unknown transmission characteristics of a future pandemic were modelled using three reproduction numbers R, with R = 1.5, 1.8, and 2.5 used in this study to capture influenza pandemics with low, medium, and high transmission characteristics, respectively. Previous pandemics of the 20^th^ century have been estimated to have had reproduction numbers in this range, with the 1918/1919 pandemic estimated as above R = 2.0 [19]–[22], the 1957 and 1968 pandemics estimated to be in the range R = 1.5 to 2.0 [19], [23], [24], while the 2009/2010 pandemic was estimated in the range R = 1.2 to 1.5 [25]–[27].

A detailed description of the model can be found in the accompanying Text S1 and in the following publications [9], [12]–[14].

### Definition of Severity

Six severity categories were defined based on the five proposed by the CDC [6], together with the addition of a very low severity category to reflect the 2009 pandemic. The CDC pandemic index was designed to better forecast the health impact of a pandemic, based on 5 categories having CFRs ranging from <0.1% to > =  2·0%. The CDC categories were extended to further include rates of hospitalisation and ICU treatment, using data collected during the 2009/2010 H1N1 pandemic in Western Australia, by the state Department of Health. These data permitted case hospitalisation (ICU and non-ICU) and case fatality ratios (CFR) to be related. These hospitalization-to-fatality and ICU-to-fatality ratios appear in Table 1, with specific CFR values for each severity category.

**Table 1:** Model parameters and cost data.

Parameters	Values	Source
Epidemiological parameters		
Symptomatic infectiousness timeline	0.5 day latent (non-infectious), 1 day asymptomatic; 2 days peak symptomatic; 2.5 days post-peak	[68]
Asymptomatic Infectiousness timeline	0.5 day latent; 5.5 days asymptomatic	[68]
asymptomatic infectiousness	0.5	[68]
peak symptomatic infectiousness	1.0	-
post-peak symptomatic infectiousness	0.5	[68]
Probability of asymptomatic infection	0.32	[17]
Probability withdrawal if symptomatic	0.5 (0.25, 0.75)* for adult; 0.9 (0.5, 1.0)* for child	-
Pandemic Severity Categories		
Severity Category 0	Case Fatality Rate = 0.03%	
Severity Category 1 (CFR<0.1%)	Case Fatality Rate = 0.1%	[6]
Severity Category 2 (CFR 0.1%–0.5%)	Case Fatality Rate = 0.25%	[6]
Severity Category 3 (CFR 0.5%–1.0%)	Case Fatality Rate = 0.75%	[6]
Severity Category 4 (CFR 1.0%–2.0%)	Case Fatality Rate = 1.5%	[6]
Severity Category 5 (CFR> = 2.0%)	Case Fatality Rate = 2.5%	[6]
Hospitalisation/fatality ratio	32:1	
ICU/fatality ratio	3:1	
Average hospital stay (days)	4 (7 for ICU hospitalisations)	[32], [69]
Antiviral parameters		
AVE - Infectiousness reduction	66% (11%, 33%)*	[54], [70]
AVE - Susceptibility reduction	85% (14%, 42%)*	[54], [70]
Prophylaxis symptom reduction probability	50%	[54]
Diagnosis delay	12 h	-
Proportion of symptomatic cases diagnosed	50%	-
AV stockpile sizes		
Treatment-only (T)	4,109 courses; 14% of population	
Treatment and Household prophylaxis (T+H)	8,805 courses; 30% of population	
T+H and Extended prophylaxis (T+H+E)	12,327 courses; 42% of population	
Social distancing parameters		
School Closure durations	2 weeks, 8 weeks and continuously	-
School Closure trigger	20–40 community cases	[13]
School Closure withdrawal probability	1.0 (0.5, 0.75)*	-
Workforce Reduction durations	4 weeks and continuously	-
Workforce Reduction attendance probability	0.5 (0.25, 0.75)*	-
Community Contact Reduction (CCR) durations	4 weeks and continuously	-
CCR and Workforce reduction trigger	2 weeks after first case	-
CCR reduction in daily contacts	0.5 (0.25, 0.75)*	-
Cost analysis assumptions		
Average wages (per week)	$836	[38]
Average school closure cost (per student per day)	$19.22	[61]
Average GP visit cost	$106.97	[37]
Average hospitalization cost (per day)	$1042	[37]
Average ICU cost (per day)	$2084	[36], [37]
Antiviral cost per course	$24.81	[37]
Antiviral dispensing cost per course	$31.22	[37]
Antiviral shelf life	5 years	[71]
Mean time between pandemics	30.3 years	-
Discount rate (annually)	3%	[31]
Average Life-expectancy in years		
0–5 age group	76.2 years	
6–17 age group	67.9 years	
18–64 age group	39.7 years	
65+ age group	14.9 years	

* Parameter range used in sensitivity analyses is given in parentheses

The simulation model determines which individuals become infected following pairwise contact of susceptible and infectious individuals, as described in the accompanying Text S1 and in the following publications [9], [12]–[14]. Our definition of case is taken to mean an infected individual who exhibits symptoms and an assumption is made that 32% of all infections are asymptomatic (see Table 1). Therefore, overall infection rates are greater than (symptomatic) attack rates and infection fatality rates (IFR) are smaller than the corresponding case fatality rates (CFR). In all results presented, severity categories are labelled by both CFR and IFR.

The CDC has defined severity categories as ranges, such as category 1 having a CFR < 0.1%, category 2 having a CFR between 0.1% and 0.5% etc [6]. For each of the five CDC severity ranges a single representative CFR was selected, with category 1 having a CFR of 0.1% and category 5 having a CFR of 2.5%, as presented in Table 1. Recent analysis of data from the 2009 pandemic suggest a CFR for the 18–64 age group in the range 0.018%–0.159%, with a median estimate of 0.029% [28]. This data was used to select a CFR for an additional severity category, namely category 0 having a CFR of 0.03%. This category was introduced to represent very low severity pandemics similar to that which occurred in 2009.

### Intervention Strategies

For each of the 6 severity categories a comprehensive range of intervention strategies including school closure, antiviral drugs for treatment and prophylaxis, workplace non-attendance (workforce reduction), and community contact reduction were examined. These interventions were considered individually and in combination and social distancing interventions were considered for either continuous periods (that is, until the local epidemic effectively ceased) or for periods of fixed duration (2, 4 or 8 weeks). The potential effectiveness of such intervention strategies in reducing the illness attack rate has been determined previously; assumptions made when modelling interventions are given in Table 1; see [9], [12]–[14] for a detailed description and rationale for these assumptions.

Antiviral and social distancing interventions were initiated when specific threshold numbers of symptomatic individuals were diagnosed in the community, and this triggered health authorities to activate the intervention response. This threshold was taken to be 0.1% of the population. It was assumed that 50% of all symptomatic individuals were diagnosed, and that this diagnosis occurred at the time symptoms appeared. Note that by “diagnosis” we do not necessarily mean laboratory confirmed diagnosis; merely that an individual sought medical attention.

Three antiviral drug strategies have been examined; antiviral drugs used solely for treatment of symptomatic cases (strategy T), T plus prophylaxis of all household members of a symptomatic case (strategy T+H), and T+H plus prophylaxis applied to the extended contact group (such as school or workplace contacts) of a symptomatic case (strategy T+H+E). Antiviral treatment (and prophylaxis for household or work/school group contacts) was assumed to begin 24 hours after the individual became symptomatic. It was assumed that an individual would receive at most one prophylactic course of antiviral drugs. Further details of antiviral interventions are given in [12], [14].

For continuous school closure, all schools were closed simultaneously once the intervention trigger threshold was reached. For fixed duration (e.g. 2 weeks or 8 weeks) school closure, schools were closed individually as follows: for a primary school the whole school was closed if 1 or more cases were detected in the school; in a high school only the class members of the affected class were isolated (sent home and isolated at home) if no more than 2 cases were diagnosed in a single class; however if there were more than 2 cases diagnosed in the entire high school the school was closed. Note that these school closure policies were only activated after the community-wide diagnosed case threshold was reached; cases occurring in schools before this time did not result in school closure. This policy of triggering school closure based on epidemic progression avoids premature school closure which can reduce the effectiveness of limited duration school closure; see [13] for a detailed description of proposed school closure strategies.

School closure (SC) was modelled by assuming home isolation such that when the intervention was in effect all school children stayed at home and did not make contact with children outside the home, and that at least one supervising adult from each affected household also stayed at home for children 12 an under. Workforce reduction (WR) was modelled by assuming that for each day the intervention was in effect each worker had a 50% probability of staying at home and thus did not make contact with co-workers. Community contact reduction (CCR) was modelled by assuming that on days when the intervention was in effect all individuals made 50% fewer random community contacts. Further details of antiviral interventions are given in [12], [14].

In the present study we simulated a total of 32 intervention scenarios. To simplify the presentation of results, only those interventions which reduce the unmitigated illness attack rate by at least 50% are reported on. A complete description of the interventions strategies is given in Text S1.

### Health Outcomes

Calculating productivity losses due to death and hospitalisation requires individual health outcomes (illness, hospitalisation, ICU admission, and death) be estimated for each severity category. Pandemic data from Western Australia provided a relationship of non-ICU hospitalisation to fatality ratio of 32:1, and an ICU admission to fatality ratio of 3:1. These values align with those published in a study by Presanis et al [29], which reported on 2009/2010 influenza pandemic statistics for the USA.

### Economic Analysis

A costing model was used to determine the total economic cost to society incurred during a pandemic, following the cost effective analysis methodology given by Drummond et al [30]. This economic model translated the daily infection profile (susceptible, infectious and recovered/immune) of each individual in the modelled population, as determined by the simulation model, into an overall pandemic cost. The overall cost comprised: costs arising from interventions (social distancing and antiviral costs); costs associated with hospitalisation of ill individuals; and productivity losses due to illness and death. Productivity losses due to death were discounted at 3% annually [31]. Life years saved in future years following the pandemic were discounted at the same rate as costs (3% per annum).

Productivity losses due to illness and interventions were calculated according to a human capital approach, using average wages (Table 1) and number of work-days lost; the latter determined using day-to-day outbreak data generated by the model.

Antiviral costs consist of two components; the cost of creating and maintaining an antiviral stockpile, and the cost of dispensing antivirals during the pandemic. We assumed that the particular antiviral regimen (either treatment; treatment and household prophylaxis; or treatment, household and extended prophylaxis) of any intervention strategy would be determined a priori as part of the pandemic planning process, that is, prior to a pandemic, and that a stockpile sufficient to carry out that particular strategy would be established and maintained.

Antiviral stockpiles sizes for each of the three antiviral regimens were calculated as the (maximum) number of antiviral courses required when each regimen is used as the sole intervention, assuming a pandemic with a reproduction number R of 1.8. If a future pandemic had a lower transmissibility than R = 1.8 (as occurred in 2009) the stockpile would be more than adequate given the assumptions made in the model, but would not be large enough for pandemics with higher transmissibility rates and thus a reproduction number greater than 1.8. The required stockpile of antiviral courses, expressed as a percentage of the population size, is 14%, 30% and 42% for treatment only, treatment and household prophylaxis, and treatment, household and extended prophylaxis regimens respectively.

Stockpile creation and maintenance costs were calculated by multiplying the cost of one antiviral course by the stockpile size and by the frequency of expiration and replacement between pandemics, assuming a mean inter-pandemic period of 30.3 years (based on pandemics in 1918, 1957, 1968 and 2009) and an antiviral shelf life of 5 years. An additional dispensing cost was included for each course of antivirals actually used during the pandemic. These costs together with stockpile sizes for the three antiviral strategies appear in Table 1.

Hospitalisation costs were determined by multiplying average cost per day by average length of stay for each age group [32]–[34]. Hospitalisation costs and costs involving medical practitioner visits are taken from the literature [35]–[37]; values are given in Table 1.

Indirect production losses due to death were based on the net present value of future earnings for an average age person in each age group, calculated by multiplying age-specific numbers of pandemic deaths by the average expectancy in years of future earnings of that age group by an average annual income, assuming an earning period to age 65 [33], [38].

There are alternative recommendations as to whether death related productivity losses should be included in cost effective analyses that take a societal perspective of costs. US standard practice excludes death related productivity losses as costs when the health outcome quantity (the denominator of the cost/effectiveness ratio) includes a length of life component [39]. The approach taken here is non-standard from a US perspective. To provide an alternative economic analysis which aligns with the US recommendations, total costs and cost effectiveness ratios for all intervention strategies omitting productivity losses due to pandemic-related deaths were also derived and appear in Text S1 (Table S1.1).

### Cost effectiveness

The cost effectiveness of a given intervention strategy is presented in terms of the cost per Life Years Saved (LYS) ratio, following methods described by Drummond et al. [30] The numerator was derived from the total cost arising from a given intervention being applied to the whole community, for each of the six severity categories. The denominator was calculated as the difference between years of life lost as a result of an unmitigated pandemic and those lost due to a pandemic with similar characteristics, but with the intervention applied. Years of life lost were derived for each simulation from the ages and life expectancies of individuals who died as a consequence of influenza infection.

The cost effectiveness of each intervention is presented as a cost in US dollars per life year saved per person. This was derived by establishing the cost effectiveness (total cost divided by total life years saved) for each intervention scenario and then dividing it by the population of the model (∼ 30,000), thus allowing the results to be applied to a population of any size.

## Results

**Figure 1:**
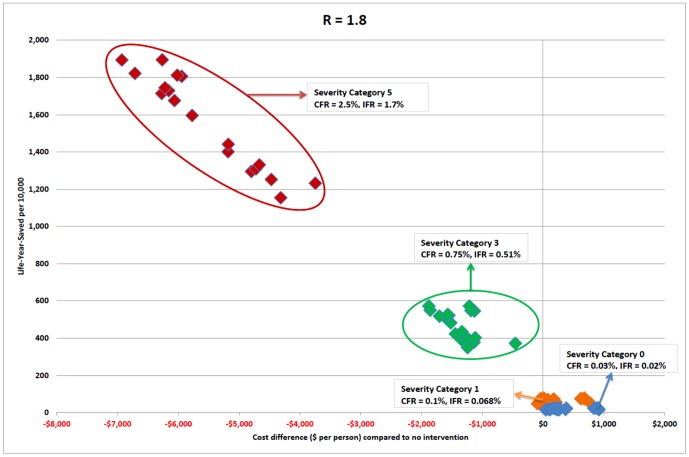
Intervention strategy cost effectiveness plane for severity categories 0, 1, 3 and 5 with death related productivity losses. Cost effectiveness for each intervention strategy is plotted as a point in a two-dimensional plane, with points coloured by severity category: blue points - category 0 (case fatality ratio 0.03%), orange points - category 1 (case fatality ratio 0.1%), green points - category 3 (case fatality ratio 0.75%), red points - category 5 (case fatality ratio 2.5%). Horizontal axis represents the cost of the intervention strategy as a difference in total cost between two scenarios; an outbreak with the intervention in place and an outbreak with no interventions, expressed as dollars per member of the population. Vertical axis represents the number of life years saved by each strategy: the difference in life years lost for an outbreak with and without the strategy in place, expressed as life years saved per 10,000 population members. Both costs (death related productivity losses) and LYS are discounted.

**Figure 2:**
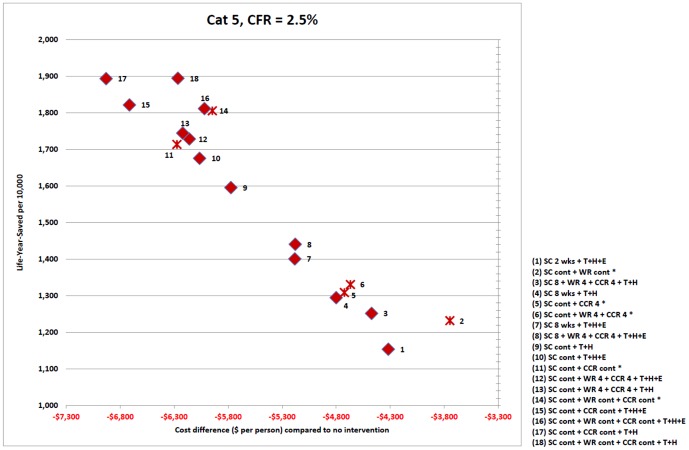
Intervention strategy cost effectiveness plane for severity category 5 with death related productivity losses. Cost effectiveness for each intervention strategy is plotted as a point in a two-dimensional plane for severity category 5 (case fatality ratio 2.5%). Horizontal axis represents the cost of the intervention strategy as a difference in total cost between two scenarios; an outbreak with the intervention in place and an outbreak with no interventions, expressed as dollars per member of the population. Vertical axis represents the number of life years saved by each strategy: the difference in life years lost for an outbreak with and without the strategy in place, expressed as life years saved per 10,000 population members. Both costs (death related productivity losses) and LYS are discounted. Strategies involving antiviral drugs are marked as diamonds; purely social distancing strategies are indicated with a cross. Each strategy is numbered, with the intervention components and durations making up the strategy given in the legend within the figure. Interventions are abbreviated as follows: SC – school closure; CCR – 50% community contact reduction; WR – 50% workforce reduction; 4, 8 – intervention duration in weeks; cont – continuous duration; T – antiviral treatment of diagnosed symptomatic cases; H – antiviral prophylaxis of household members of diagnosed symptomatic cases, E – antiviral prophylaxis of school class or workplace contacts of diagnosed symptomatic cases.

**Figure 3:**
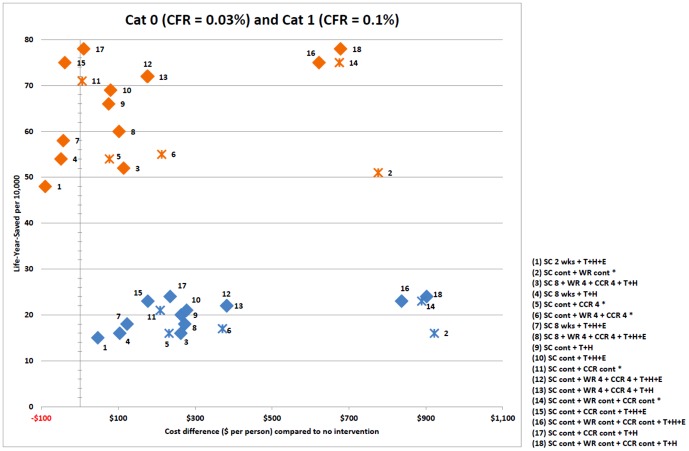
Intervention strategy cost effectiveness plane for severity category 0 and category 1 with death related productivity losses. Cost effectiveness for each intervention strategy is plotted as a point in a two-dimensional plane for severity category 0 (case fatality ratio 0.03%) (blue points) and category 1 (case fatality ratio 0.1%) (orange points). Horizontal axis represents the cost of the intervention strategy as a difference in total cost between two scenarios; an outbreak with the intervention in place and an outbreak with no interventions, expressed as dollars per member of the population. Vertical axis represents the number of life years saved by each strategy: the difference in life years lost for an outbreak with and without the strategy in place, expressed as life years saved per 10,000 population members. Both costs (death related productivity losses) and LYS are discounted. Strategies involving antiviral drugs are marked as diamonds; purely social distancing strategies are indicated with a cross. Each strategy is numbered, with the intervention components and durations making up the strategy given in the legend within the figure. Interventions are abbreviated as follows: SC – school closure; CCR – 50% community contact reduction; WR – 50% workforce reduction; 4, 8 – intervention duration in weeks; cont – continuous duration; T – antiviral treatment of diagnosed symptomatic cases; H – antiviral prophylaxis of household members of diagnosed symptomatic cases, E – antiviral prophylaxis of school class or workplace contacts of diagnosed symptomatic cases.

Results are presented as follows: Figure 1 captures the relative cost effectiveness of intervention strategies, for severity categories 0, 1, 3 and 5. Figure 2 pictures intervention cost effectiveness for severity category 5, and Figure 3 severity categories 0 and1, in greater detail. Table 2 shows the number of life years saved and cost per life year saved for each strategy and severity category. Table 3 presents the breakdown of costs attributed to each strategy for categories 1 and 5; and Table 4 presents the number of lives saved and hospitalisations avoided due to each intervention strategy.

**Table 2:** Intervention strategy, cost effectiveness (with death related productivity losses) and life years saved.

Intervention Strategy	AR	Cat 0	Cat 1	Cat 2	Cat 3	Cat 4	Cat 5
	(%)	CFR = 0.03%	CFR = 0.1%	CFR = 0.25%	CFR = 0.75%	CFR = 1.5%	CFR = 2.5%
		IFR = 0.02%	IFR = 0.068%	IFR = 0.17%	IFR = 0.51%	IFR = 1.02%	IFR = 1.7%
no intervention	32	-	-	-	-	-	-
		-	-	-	-	-	-
SC 2 wks+T+H+E	15	$155,516	$73,650	$50,917	$40,411	$37,772	$36,695
		15	48	116	349	693	1154
*SC cont+WR cont	15	$709,622	$238,774	$115,953	$58,933	$44,739	$38,994
		16	51	124	372	740	1232
SC 8+WR 4+CCR 4+T+H	14	$280,148	$107,277	$61,350	$40,056	$34,742	$32,585
		16	52	126	378	752	1252
SC 8 wks+T+H	14	$173,457	$73,085	$45,942	$33,374	$30,229	$28,950
		16	54	130	391	778	1295
*SC cont+CCR 4	14	$249,088	$95,606	$54,789	$35,866	$31,143	$29,225
		16	54	132	396	786	1309
*SC cont+WR 4+CCR 4	13	$328,138	$118,862	$63,713	$38,129	$31,751	$29,165
		17	55	134	402	800	1331
SC 8 wks+T+H+E	12	$170,942	$68,609	$41,193	$28,490	$25,315	$24,026
		18	58	141	423	842	1401
SC 8+WR 4+CCR 4+T+H+E	11	$248,891	$91,104	$49,475	$30,164	$25,349	$23,397
		18	60	145	435	866	1441
SC cont+T+H	9	$220,269	$78,133	$40,789	$23,461	$19,143	$17,394
		20	66	161	482	959	1596
SC cont+T+H+E	8	$216,534	$75,083	$38,035	$20,840	$16,558	$14,823
		21	69	169	506	1007	1676
*SC cont+CCR cont	7	$179,889	$63,100	$32,466	$18,250	$14,708	$13,273
		21	71	173	518	1029	1714
SC cont+WR 4+CCR 4+T+H+E	7	$257,440	$86,377	$41,775	$21,068	$15,914	$13,827
		22	72	174	522	1039	1729
SC cont+WR 4+CCR 4+T+H	7	$255,140	$85,337	$41,080	$20,532	$15,417	$13,347
		22	72	176	527	1048	1745
*SC cont+WR cont+CCR cont	6	$469,517	$149,344	$66,397	$27,869	$18,288	$14,413
		23	75	182	546	1085	1806
SC cont +CCR cont+T+H+E	6	$154,908	$53,302	$26,726	$14,391	$11,319	$10,075
		23	75	183	550	1094	1822
SC cont+WR cont+CCR cont+T+H+E	6	$444,971	$141,754	$63,194	$26,704	$17,630	$13,961
		23	75	183	548	1089	1812
SC cont+CCR cont+T+H	5	$173,483	$57,626	$27,455	$13,447	$9,961	$8,550
		24	78	191	572	1138	1894
SC cont+WR cont+CCR cont+T+H	5	$453,160	$142,730	$62,401	$25,087	$15,809	$12,058
		24	78	191	572	1138	1895

Final symptomatic attack rate (AR), cost per LYS (bold) and life-years saved (LYS) are shown for each intervention strategy and each severity category. LYS expressed as years per 10,000 members of population. Both costs (death related productivity losses) and LYS are discounted. Values are for pandemic with unmitigated transmissibility of R_0_ = 1.8. Interventions abbreviated as: SC – school closure; CCR – 50% community contact reduction; WR – 50% workforce reduction; 4, 8 – intervention duration in weeks; cont – continuous duration; T – antiviral treatment of diagnosed symptomatic cases; H – antiviral prophylaxis of household members of diagnosed symptomatic cases, E – antiviral prophylaxis of school class or workplace contacts of diagnosed symptomatic cases. Pure social distancing interventions marked by *.

**Table 3:** Total pandemic costs (with death related productivity losses), cost breakdowns and percentages of different cost components.

	Category 1 (CFR 0.1%)					Category 5 (CFR 2.5%)				
Interventions	Total Cost	HCC	AVC	ISDPL	DRPL	Total Cost	HCC	AVC	ISDPL	DRPL
no intervention	$441	$86	$0	$65	$290	$8,550	$1,472	$0	$65	$7,013
		19%	0%	15%	66%		17%	0%	1%	82%
SC 2 wks+T+H+E	$351	$41	$74	$97	$139	$4,233	$704	$74	$97	$3,358
		12%	21%	28%	40%		17%	2%	2%	79%
*SC cont+WR cont	$1,217	$39	$0	$1,050	$128	$4,804	$661	$0	$1,050	$3,092
		3%	0%	86%	11%		14%	0%	22%	64%
SC 8+WR 4+CCR 4+T+H	$555	$37	$50	$342	$126	$4,079	$638	$50	$342	$3,048
		7%	9%	62%	23%		16%	1%	8%	75%
SC 8 wks+T+H	$392	$36	$50	$186	$120	$3,750	$614	$50	$186	$2,900
		9%	13%	47%	31%		16%	1%	5%	77%
*SC cont+CCR 4	$518	$36	$0	$364	$118	$3,826	$612	$0	$364	$2,849
		7%	0%	70%	23%		16%	0%	10%	74%
*SC cont+WR 4+CCR 4	$654	$35	$0	$505	$115	$3,882	$597	$0	$505	$2,781
		5%	0%	77%	18%		15%	0%	13%	72%
SC 8 wks+T+H+E	$398	$32	$73	$187	$106	$3,367	$543	$73	$187	$2,565
		8%	18%	47%	27%		16%	2%	6%	76%
SC 8+WR 4+CCR 4+T+H+E	$543	$30	$72	$340	$101	$3,371	$514	$72	$340	$2,445
		6%	13%	63%	19%		15%	2%	10%	73%
SC cont+T+H	$516	$24	$48	$363	$81	$2,775	$418	$48	$363	$1,946
		5%	9%	70%	16%		15%	2%	13%	70%
SC cont+T+H+E	$521	$21	$70	$359	$70	$2,485	$364	$70	$359	$1,691
		4%	13%	69%	13%		15%	3%	14%	68%
*SC cont+CCR cont	$447	$20	$0	$363	$65	$2,275	$337	$0	$363	$1,575
		4%	0%	81%	15%		15%	0%	16%	69%
SC cont+(WR+CCR) 4+T+H+E	$618	$19	$69	$466	$63	$2,390	$324	$69	$466	$1,531
		3%	11%	75%	10%		14%	3%	20%	64%
SC cont+WR 4+CCR 4+T+H	$616	$12	$22	$1,045	$41	$2,329	$314	$47	$489	$1,478
		1%	2%	93%	4%		13%	2%	21%	63%
*SC cont+WR cont+CCR cont	$1,116	$16	$0	$1,047	$53	$2,603	$273	$0	$1,047	$1,284
		1%	0%	94%	5%		10%	0%	40%	49%
SC cont+CCR cont+T+H+E	$402	$15	$68	$267	$51	$1,835	$260	$68	$267	$1,240
		4%	17%	66%	13%		14%	4%	15%	68%
(SC+WR+CCR) cont+T+H+E	$1,063	$15	$68	$926	$53	$2,530	$263	$68	$926	$1,272
		1%	6%	87%	5%		10%	3%	37%	50%
SC cont+CCR cont+T+H	$452	$12	$46	$351	$42	$1,619	$214	$46	$351	$1,008
		3%	10%	78%	9%		13%	3%	22%	62%
(SC+WR+CCR) cont+T+H	$1,119	$12	$46	$1,018	$42	$2,284	$210	$46	$1,018	$1,009
		1%	4%	91%	4%		9%	2%	45%	44%

Total costs (bold) are expressed as dollars per member of population. Cost breakdowns are also expressed as dollars per member of population. Percentages of cost components of the total cost for each intervention are presented in cells below the corresponding cost. Death related productivity losses are discounted. Cost categories are abbreviated as follows: HCC – health care costs (GP visits, hospitalisation and ICU usage), AVC – antiviral costs (cost of drugs, dispensing and stockpile renewal), ISDPL – illness and social distancing productivity losses, DRPL – death-related productivity losses. Interventions abbreviated as: SC – school closure; CCR – 50% community contact reduction; WR – 50% workforce reduction; 4, 8 – intervention duration in weeks; cont – continuous duration; T – antiviral treatment of diagnosed symptomatic cases; H – antiviral prophylaxis of household members of diagnosed symptomatic cases, E – antiviral prophylaxis of school class or workplace contacts of diagnosed symptomatic cases. Pure social distancing interventions are marked by *.

**Table 4:** Life-Years saved, death averted and hospitalisation averted.

	Cat 1, CFR = 0.1%			Cat 5, CFR = 2.5%		
Intervention strategy	LYS	Deaths averted	Hospitalisations averted	LYS	Deaths averted	Hospitalisations averted
no intervention	0	0	0	0	0	0
SC 2 wks+T+H+E	48	1	56	1154	42	1366
*SC cont+WR cont	51	1	59	1232	45	1442
SC 8+WR 4+CCR 4+T+H	52	2	61	1252	46	1482
SC 8 wks+T+H	54	2	63	1295	47	1525
*SC cont+CCR 4	54	2	63	1309	47	1527
*SC cont+WR 4+CCR 4	55	2	64	1331	48	1556
SC 8 wks+T+H+E	58	2	68	1401	51	1651
SC 8+WR 4+CCR 4+T+H+E	60	2	70	1441	53	1703
SC cont+T+H	66	2	77	1596	58	1872
SC cont+T+H+E	69	2	81	1676	61	1968
*SC cont+CCR cont	71	2	83	1714	62	2016
SC cont+(WR+CCR) 4+T+H+E	72	2	84	1729	63	2039
SC cont+WR 4+CCR 4+T+H	72	2	85	1745	64	2057
*SC cont+WR cont+CCR cont	75	2	88	1806	66	2131
SC cont+CCR cont+T+H+E	75	2	89	1822	67	2153
(SC+WR+CCR) cont+T+H+E	75	2	89	1812	67	2148
SC cont+CCR cont+T+H	78	3	92	1894	69	2236
(SC+WR+CCR) cont+T+H	78	3	93	1895	69	2241

Life-years saved (LYS), deaths averted and hospitalisations averted are shown per 10,000 members of population for each intervention strategy and for severity category 1 and 5. Values are for pandemic with unmitigated transmissibility of R_0_ = 1.8. Life-years saved (LYS) are discounted. Interventions abbreviated as: SC – school closure; CCR – 50% community contact reduction; WR – 50% workforce reduction; 4, 8 – intervention duration in weeks; cont – continuous duration; T – antiviral treatment of diagnosed symptomatic cases; H – antiviral prophylaxis of household members of diagnosed symptomatic cases, E – antiviral prophylaxis of school class or workplace contacts of diagnosed symptomatic cases. Pure social distancing interventions marked by *.

The cost effectiveness for each intervention strategy is plotted as a point in a two-dimensional plane in Figures 1, 2 and 3. The horizontal axis represents the overall cost of the strategy, as the difference in cost when comparing that of an unmitigated epidemic with that with the intervention in place, expressed as the total cost in dollars per member of the population. The vertical axis represents the number of life years saved arising from each intervention strategy; that is, the difference in the number of life years lost between an outbreak with and without that intervention strategy activated, expressed as life years saved per 10,000 population members.

Figure 1 presents the cost effectiveness of a comprehensive range of social distancing and antiviral intervention strategies for four severity categories: category 0 (case fatality ratio 0.003%, blue points); category 1 (case fatality ratio 0.1%, orange points), category 3 (case fatality ratio 0.75%, green points), and category 5 (case fatality ratio 2.5%, red points).

For each of the four severity categories pictured in Figure 1, the same set of interventions are applied to a pandemic with transmissibility corresponding to reproduction number R = 1.8, and a related final symptomatic attack rate of 32%. The relative cost effectiveness of each intervention is reflected by the placement of each intervention in the two dimensional cost effectiveness plane. The effect of severity on the placement can be readily seen in Figures 1, 2 and 3. These three figures present results for 18 effective intervention strategies, those which reduce the attack rate by at least 50%. Table 2 presents cost effectiveness results for all severity categories and the 18 effective interventions. Text S1 (Table S1.12) gives the results for all 29 intervention strategies which were examined, including those (such as sole social distancing or antiviral interventions) which have limited attack rate reduction effectiveness.

### Cost Effectiveness Trends

Certain trends are apparent in Figure 1 and these show that severity is a strong determining factor in the cost effectiveness of interventions. The highest severity interventions appear in the top left of Figure1 and the lowest at the bottom right. This indicates that the cost of saving a life for each of the four severities pictured is higher for low severity pandemics compared to high severity pandemics. The cost per life year saved is substantial for low severity pandemics (categories 0 and 1), as presented in Table 2.

The number of life years saved by each intervention strategy (the vertical axis) increases with severity. This is unsurprising since severity is determined by the case fatality ratio, the proportion of symptomatic cases resulting in death. As interventions reduce the number of infections and thus symptomatic cases, so an intervention which reduces the attack rate by a particular amount will result in a greater number of life years saved for a high severity pandemic compared to one of low severity.

This can be seen in Figure 1 for category 0 (blue points) and 1 (orange points) pandemics, which by definition have inherently low unmitigated case mortality rates, of 0.03% and 0.1% respectively. The number of life years saved by interventions is also low and occurs in a narrow vertical LYS band, showing that there is little difference in life years saved between the different intervention strategies for these low and very low severity categories.

In contrast to low severity pandemics, a category 5 pandemic (red points) has a high unmitigated mortality rate and a substantial number of life years are saved by effective interventions. Figure 1 illustrates the large vertical spread in life years saved between the most effective and least effective strategies for category 5 pandemics, highlighting the fact that some interventions are substantially more cost effective than others.

For high severity pandemics, the cost savings resulting from interventions closely correlates with the number of life years saved. The most effective interventions, in terms of attack rate and consequential death reductions, are also the most cost effective. This relationship can be seen in Figure 1, by the linear shaped grouping, running from top left to bottom right, of the red points representing category 5 (high severity); illustrated in greater detail in Figure 2. The greater the number of life years saved by an intervention strategy, the greater the cost savings. This is due to the fact that for high severity pandemics, the majority of costs stem directly from both death and illness related productivity losses and from hospitalisation and ICU costs, all of which are proportional to the number of deaths. Avoided deaths thus give rise to cost savings compared to the no-intervention baseline, and these savings are larger at higher severities as more deaths are prevented. This can be seen in Table 3, which compares the breakdown of total cost components, and in Table 4, which compares the number of deaths and hospitalisations avoided for severity categories 1 and 5. Avoided deaths thus give rise to both life years saved and cost savings.

By contrast, low and very low severity pandemics of category 1 and 0 respectively, as shown in detail in Figure 3, have no strong correlation between life years saved and intervention cost. This is due to the small cost savings from (the few) avoided deaths in comparison to direct intervention costs, specifically those arising from productivity losses due to school and workplace closure; see Table 3 for cost breakdowns and Table 4 for the number of deaths and hospitalisations avoided. The same social distancing costs exist for pandemics of higher severity, but they are fixed and do not increase with severity. Hence intervention-related social distancing costs are exceeded by severity-related costs, such as increased hospitalisation costs and illness and death related productivity losses, at higher severity categories.

Below we report on the cost effectiveness of interventions strategies for pandemics of high and low severity. These data are given in Table 2, which presents the final attack rate (AR), the number of life years saved per 10,000 person population and the cost effectiveness, as cost per person per life year saved, for each intervention strategy. Interventions are ordered from top to bottom by increasing effectiveness, in terms of their ability to decrease the attack rate, and only the 18 intervention strategies which reduce the attack rate by at least 50% are included.

Text S1 (Table S1.12) includes all 29 intervention strategies examined by this study. The additional interventions listed in this table have attack rates of 16% and greater. For all of these interventions, and for all severity categories, less costly interventions exist which have attack rates less than 16%. For categories 0, 1 and 2, the maximal use of antivirals without social distancing interventions (treatment and household and extended contact prophylaxis) is the most cost effective intervention of those with attack rates greater than 16%. To gain further life years from this intervention strategy requires reducing the attack rate further, which is achieved by coupling this intervention with sustained social distancing interventions, especially school closure.

### High Severity Pandemics

For a high severity pandemic (category 5, case fatality ratio 2.5%) the cost effectiveness of interventions ranges from $8,550 to $38,994 per life year saved, resulting in 1154 to 1895 life years saved (per 10,000 person population), as in Figure 2 and Table 2.

For all severity categories, a strategy which combines continuous school closure, community contact reduction, and antiviral treatment and household prophylaxis gives rise to the largest number of life years saved. For pandemics with severity categories 3 and above (CFR > =  0.75%) this strategy also has the lowest total cost, and is therefore more cost effective relative to all other strategies. This strategy is the most effective and most cost effective available and is shown for pandemic category 5 as point 17 in Figure 2, which lies both above (giving more life years saved) and to the left (having a lower cost) of all other interventions including the origin, which represents the no intervention scenario.

The least cost effective strategy considered, which still reduced the attack rate by at least 50%, is continuous school closure combined with continuous workforce reduction. It is the presence of workforce reduction which causes this to be less cost effective than other strategies; it is costly in terms of productivity lost and not very effective in reducing the attack rate and subsequent mortality rate

The fact that the intervention strategies which are most effective at saving lives are also the most cost effective for high severity pandemics is explained by the fact that for severe pandemics the major pandemic cost arises from death-related productivity losses and hospitalisation costs. Interventions which prevent the most infections and save the most lives therefore also reduce the largest component of the total pandemic cost. The different cost components which contribute to the total cost of an intervention strategy are presented in Table 3, for category 1 and 5 pandemics.

### Low Severity Pandemics

For a low severity pandemic (category 1, with case fatality ratio 0.1%) the cost effectiveness of intervention strategies ranges from $53,302 to $238,774 per life year saved, which results in 153 to 105 life years saved (per 10,000 population) respectively; see Figure 3 and Table 2.

For a low severity category 1 pandemic the least costly intervention considered was two weeks school closure combined with antiviral treatment and household and extended prophylaxis. The low cost of this intervention results from the use of minimal social distancing resources to reduce the work days lost due to illness. In contrast to higher severity pandemics, this least costly strategy is not the most cost effective, costing $73,650 per life year saved; see Table 2. It is also less effective in reducing the attack rate and saves only 48 life years (per 10,000 population).

The most effective strategy in terms of attack rate reduction involves combining continuous school closure and community contact reduction with antiviral treatment and household prophylaxis, saving 78 life years (per 10,000 population) at a cost of $57,626 per life year saved. The most cost effective strategy adds extended prophylaxis to this strategy, saving almost the same number of life years (75) at a cost of $53,302 per life year saved. Compared to the least costly strategy, the most effective strategy saves an additional 30 life years (per 10,000 population).

For a category 1 pandemic the orange cluster of most costly intervention strategies (numbered 2, 14, 16 and 18 on the top, right-hand side of Figure 3) all involve continuous workforce reduction. These are the least cost effective strategies, at $141,754 to $238,774 per life year saved. For category 0, 1 and 2 pandemics, while the strategies which involve continuous workforce reduction reduce medical costs and death-related productivity losses, they incur relatively larger costs due to lost productivity, resulting in costs per life year saved which are higher than all other strategies.

### Very Low Severity Pandemics

For a pandemic with very low severity (category 0, with case fatality ratio 0.03%) similar to the 2009 pandemic, and in the range 0.005%–0.07% estimated for seasonal influenza epidemics [40], [41], all intervention strategies are costly and none may be seen to be highly cost effective. Even the most cost effective strategy (continuous school closure and community contact reduction coupled with the maximum antiviral interventions) had a cost per LYS of $154,908. The same strategy used with a category 1 pandemic had a cost of $53,302 per LYS, and significantly lower costs than this for categories 2 and above; see Table 2.

For mild category 0 pandemics, as occurred in 2009, the costs incurred by intervention measures (antiviral costs and productivity losses due to the need for child care during school closure) are significantly larger than productivity losses and medical costs averted by the interventions. This contrasts with the situation for pandemics with severity of category 1 and above (CFR of 0.1% and greater) where the costs of continuous school closure and antiviral therapy are outweighed by the savings in medical costs and death-related productivity losses, see Table 3.

### Intervention Strategies without Antiviral Use

During a future pandemic, many countries will not have access to antiviral stockpiles sufficient to implement large-scale treatment and prophylactic strategies [42]. Furthermore, a newly emerged pandemic influenza strain may develop antiviral drug resistance, rendering antiviral strategies less effective [43]–[46]. An important class of interventions are the purely social distancing strategies, which may be rapidly activated once a pandemic strain appears in a community. The most effective such strategy involves the continuous application of the combination of all social distancing interventions, namely school closure and workforce and community contact reduction. For pandemics ranging from very low to high severity in categories 0 to 5, this intervention strategy results in from 23 to 1806 life years saved (per 10,000 population) with cost effectiveness ratio from $469,517 to $14,413 per life year saved respectively; see Table 2.

The least costly non-pharmaceutical strategy omits workforce reduction from the most effective strategy given above. This strategy is slightly less effective but is more cost effective, costing from $179,889 per life year saved to $13,273 per life year saved, for severity categories ranging from 0 to 5 respectively (see Table 2). The addition of continuous workforce reduction to this strategy constitutes a cost effectiveness trade-off: for a category 5 pandemic this saves an additional 92 life years (per 10,000 population) and costs an additional $328 per person, giving. For a category 1 pandemic this trade-off is much less favourable, saving an additional 4 life years (per 10,000 population) at an additional cost of $669 per person. For a category 0 pandemic, the addition of continuous workforce reduction saves an additional 2 life years at an addition cost of $680 per person.

### Results without Death-Related Productivity Losses

The economic analysis method included future productivity losses due to pandemic related deaths. Death-related productivity losses make up a significant proportion of the cost of high severity pandemics. An alternative economic analysis consistent with US cost effective analysis standards [39], which omits death-related productivity losses, was also conducted and the detailed results are included in Text S1 (Table S1.1 and Figure S1.1).

Due to the large contribution of death-related productivity losses, the total cost of pandemics is lower if this cost is excluded, by a factor ranging from 2.0 at category 1 to 5.5 at category 5, for unmitigated pandemics.

However, for categories 3 and above, the relative cost and cost effectiveness of all strategies within a severity category are essentially unchanged when death costs are omitted, and the results are thus independent of which cost analysis method is used. For high severity pandemics (categories 3, 4 and 5), strategies which combine continuous school closure and community contact reduction with antiviral measures result are both the most effective at reducing mortality and the most cost effective. For high severity pandemics, the inclusion or exclusion of death-related productivity losses leaves the qualitative outcome of the analyses unchanged.

For low severity pandemics (categories 1 and 2), it was found that all strategies which combined school closure of any duration (with or without community contact reduction) and antiviral measures had very similar cost effectiveness ratios, between $44,000 and $66,000 per LYS for category 1 and between $19,000 and $28,000 per LYS for category 2.

For categories 0, 1 and 2, shorter durations of school closure and community contact reduction strategies are found to be as cost effective, or more cost effective, than continuous duration strategies, when coupled with antiviral interventions. Continuous social distancing interventions are more cost effective than those of limited duration for pandemics with severity of category 3 and up using both cost effective analysis methods.

### Sensitivity Analyses

Sensitivity analyses were conducted for key model parameters to determine how the cost effectiveness results are affected by variations in these parameter values. Full details of these analyses can be found in Text S1; the most significant outcomes are summarised below.

### Transmissibility

The above results are based on a pandemic with reproduction number R of 1.8. Alternative reproduction numbers of 1.5 and 2.5 were also examined, spanning a range of estimates of the transmissibility of a pandemic influenza virus strain [7], [8], [25]–[27], [47]; see Text S1 (Figure S1.2).

The primary effect of varying transmissibility was in altering the attack rate and thus the number of hospitalisations and deaths, which in turn determined medical costs and death-related productivity losses. At the lower transmissibility of R = 1.5 both the overall pandemic costs and the number of deaths was lower. Interventions saved fewer lives and as a consequence, intervention strategies were less cost effective per life year saved compared to the same interventions at higher transmissibility. The opposite was true for high transmissibility of R = 2.5 where the number of deaths and the total cost were higher, meaning that interventions were more cost effective on a life year saved basis compared to the same interventions at lower transmissibility.

A secondary effect of altering transmissibility was in the effectiveness of interventions. Lower transmissibility renders interventions more effective, and vice-versa. While this effect was observed, its impact on cost effectiveness was smaller than the direct influence of transmissibility on attack rate and mortality rates described above.

### Prior Immunity

In the main results it was assumed that individual susceptibility to infection differed by age, resulting in age-specific infection rates similar to the 2009 pandemic, where 18–24 years olds had the highest attack rates while those 25 years and older had the lowest [16]. Previous pandemics have exhibited different age-specific attack rate profiles. The 1957 pandemic resembled seasonal influenza with the highest attack rates in children, while the 1968 pandemic had similar attack rates in all age groups [8]. In a future pandemic caused by a highly novel influenza strain, it may be the case that the population has no prior immunity. To examine this possibility, the sensitivity of the main results were assessed using an alternative baseline pandemic with the same unmitigated attack rate, but where all age groups were equally susceptible.

This alternative baseline shifted the burden of illness to older age groups, and for most interventions resulted in slightly lower attack rates. The net result of these two effects was that the number of life years saved (LYS) was similar to the 2009 age-specific susceptibility baseline, in most cases being slightly lower. However, a finding consistent across all interventions and severity levels was that total pandemic costs were lower. This was due to the fact that the average age of influenza fatalities was higher, meaning that fewer working years of life were lost resulting in lower death-related productivity losses. As a result, all interventions exhibited a slightly lower cost per LYS compared to the 2009 age-specific susceptibility baseline. Results of this sensitivity analysis are contained in Text S1 (Table S1.9).

### Asymptomatic Infection

The baseline assumption was that the asymptomatic rate, the proportional of infected individuals who do not experience symptomatic infection, was 32% for adults and 20% for children, averaging 30% across all age groups. These proportions were based on studies of seasonal influenza [17]. In contrast, evidence suggests that the 2009 pandemic had a higher asymptomatic rate of approximately 60% (across all age groups) [18] and a sensitivity analysis was conducted with this alternative baseline assumption.

It was found that the higher asymptomatic rate resulted in interventions which were less cost effective compared to a pandemic with a lower asymptomatic rate but the same case fatality ratio and transmissibility. This was true across all severity categories and interventions. This lower cost effectiveness ratio is explained by the fact that the higher asymptomatic rate rendered interventions relatively less effective as schools will remain open longer prior to closure and antiviral use will be reduced. Results of this sensitivity analysis are contained in Text S1 (Table S1.10).

### Intervention Parameters

Sensitivity analyses were conducted to measure the effect of varying key parameters on the cost effectiveness of each type of intervention. These intervention parameters were: antiviral efficacy; degree of community contact reduction; compliance with home isolation during school closure; and degree of workforce reduction. For each parameter alternative values were examined, which rendered the interventions more or less effective at preventing transmission and lessening the attack rate.

#### Antiviral efficacy

If antiviral agents are assumed to have lower efficacy than the baseline, which assumes 66% infectiousness reduction when used for treatment (and 85% susceptibility reduction when used prophylactically), then intervention strategies which depend heavily on antiviral measures (i.e. antiviral-only strategies or antivirals coupled with 2 weeks school closure) were shown to be less effective and less cost effective; see Text S1 (Table S1.5) for categories 1 and 5 efficacy results.

It can be seen that for the treatment only strategy T, the attack rate increase and cost effectiveness decreases are slight when the efficacy drops to 33% (42%) and 11% (14%); however for the treatment and household/extended prophylaxis antiviral strategy T+H+E there are significant increases in attack rate (19% to 27%) and substantial decreases in cost effectiveness (by a factor of ∼ 4) when the efficacy drops to 11% (14%), for both severity categories 1 and 5. When antiviral measures were coupled with rigorous social distancing (i.e. continuous school closure) the impact of lower antiviral efficacy was found to be similar but less marked, as the antiviral component of the combined intervention strategy only contributes a part of the overall attack rate reduction.

In addition to antiviral cost effectiveness being dependent on drug efficacy for both treatment and prophylaxis, as described above, Text S1 (Table S1.5, using the costing methodology which excludes death-related losses) highlights the feature that the cost effectiveness of antiviral-based intervention strategies is also strongly dependent on pandemic severity. Taking a somewhat arbitrary figure of $50,000 per life year saved as a threshold figure for determining if a given intervention is cost effective or not, then only two strategies for category 1 pandemics fall (slightly) below this threshold. Both involve maximal (baseline) efficacy and the maximal antiviral strategy, involving use of antiviral agents for treatment, household and extended contact prophylaxis. By contrast, for category 5 pandemics all antiviral based strategies are below this $50,000 threshold except for two, and these are only slightly above it, with many of the strategies being substantially below the threshold.

#### Weaker social distancing

Lower levels of compliance to community contact reductions (25% fewer community contacts rather than 50%) resulted in less effective interventions and higher total costs, rendering strategies which included such measures less cost effective. Conversely, higher levels of compliance increased effectiveness and cost effectiveness; see Text S1 (Table S1.8).

In the case of school closure, decreases in compliance with home isolation during school closure gave rise to a small decrease in the direct cost of the intervention, as productivity losses due to parental supervision decreased. However these cost savings were small compared to indirect losses due to the consequential decrease in school closure effectiveness, which led to increased infection and illness-related productivity losses and medical costs. Higher compliance to home isolation during school closure was therefore more cost effective than lower compliance; see Text S1 (Table S1.6).

Changes in the effectiveness of workforce reduction had minimal effect on cost effectiveness, as increasing (or decreasing) levels of workforce reduction resulted in linked increases (or decreases) in both life years saved and productivity losses. Full results of these analyses are presented in Text S1 (Table S1.7).

## Discussion

An individual-based simulation model was combined with an economic analysis methodology to determine health outcomes and the cost effectiveness of interventions which would be used during future influenza pandemics. These results give a comprehensive analysis of the cost effectiveness of pandemic interventions in a developed country setting, highlight how pandemic severity impacts on pandemic costs and provide guidance in the development and refinement of pandemic preparedness plans. They should inform public health authorities as to how best to allocate intervention resources during a pandemic, and how to adjust interventions depending on emerging knowledge of pandemic severity.

For severity categories from 0 to 5, the most effective intervention strategies involve continuous school closure and community contact reduction, coupled with antiviral treatment and prophylaxis. These strategies are also the most cost effective when measured by cost per life year saved. For high severity pandemics, of category 3 and above, pandemic costs are dominated by hospitalisation costs and productivity losses due to death, and as a result the intervention strategies which save the most lives also have the lowest total cost, making those strategies more cost effective compared to less effective strategies. For low severity pandemics, the most effective strategies are still the most cost effective in terms of cost per life year saved, but there are alternative strategies that are less cost effective and which allow mortality reduction to be traded off against a reduction in total pandemic cost.

For pandemics with very low category 0 severity, having a case fatality ratio (CFR) of 0.03% similar to that of the 2009 pandemic, all strategies give rise to total costs that are higher than the no-intervention baseline, as intervention costs are not compensated by reductions in medical and productivity losses. Coupled with the fact that at very low severity few lives are saved by interventions, even the most cost effective strategies have a high cost per life year saved. Whether antivirals are really needed for “mild” (i.e. low severity) pandemics is probably a separate question to that of their cost effectiveness or even their relative cost effectiveness; if symptomatic infection rates are low, and if symptoms are generally “mild”, then the results indicate that all effective (in terms of attack rate reduction) interventions involving antivirals and/or social distancing measures are highly expensive per life year saved. So choosing to utilise them for “mild” pandemics is expensive for the benefit gained and public health authorities may wish to activate measures more to reassure the public than out of necessity. For high severity pandemics the benefits, both from a health outcome and from an (associated) cost effectiveness perspective, of using aggressive intervention strategies is clear.

Our results reiterate prior findings in terms of the necessity of combining multiple intervention measures in order to achieve substantial reductions in symptomatic case numbers and consequential mortality rates [9], [12], [13], [48]–[51]. Such combined interventions, particularly if they are sustained, are found to be highly cost effective for severe pandemics.

The different interventions which may be combined to form an intervention strategy have varying effectiveness in reducing the attack rate, and contribute differentially to the total epidemic cost and cost per life year saved. School closure and associated home quarantining always results in a reduced attack rate, with greater reductions resulting from longer duration closures. Significantly, long duration school closure is a component in all the most cost effective intervention strategies for pandemics in all severity categories 1 and above. For severity category 0, all interventions are highly costly on a cost per life year saved basis, however short-term school closure, while not highly effective at reducing the attack rate, is more cost effective than long-term closure. It was assumed that children affected by school closure would be quarantined in the home, and it was found that any decrease in the level of home isolation rendered school closure both less effective and less cost effective. Note that it was assumed that school closures were optimally timed, with schools closing according to a policy that takes into account the duration of school closure and the transmissibility of the pandemic [13].

Community contact reduction is always effective in reducing the number of deaths and in the economic analysis used model had no direct economic cost. As a result community contact reduction is found to be a cost effective addition to all intervention strategies.

For severity categories of 1 and above, and under the parameter setting used in this analysis, the use of antiviral agents as an adjunct to social distancing interventions increases their effectiveness and cost effectiveness, and all the most effective and cost effective strategies involve their use. For pandemics of very low severity in category 0, all social distancing intervention strategies have a high cost per life year saved, and the use of antivirals does not render such interventions cost effective.

The cost effectiveness of antiviral interventions were found to depend strongly on the efficacy of antivirals in reducing transmission when used for treatment and prophylaxis. Strategies that rely heavily on antivirals, such as antiviral-only strategies, or strategies where antivirals are coupled with limited duration social distancing, are far less cost effective if antiviral efficacy is substantially lower than currently assumed. Results on how reduced antiviral efficacy impacts the overall effectiveness and cost effectiveness of antiviral-based strategies, given in Text S1 (Table S1.5), highlight the importance of reliable estimates of antiviral efficacy. These estimates are currently based on limited studies [52]–[54]; the issue of whether or not current estimates are reliable is discussed in [55] and the consequences of lower efficacy in [56].

This study has made plausible assumptions regarding the logistics of antiviral interventions. Previous research has examined how logistical factors may impact the effectiveness, and thus cost effectiveness of antiviral based interventions. These logistical factors include the required stockpile size and distribution capacity [13], [57]–[59], the proportion of symptomatic individuals receiving treatment or prophylaxis [14], [60], and the delay between infection and the provision of antiviral drugs [14], [49].

In the situation where the severity of a future pandemic is unknown at its early stages, as occurred with the 2009 pandemic, a realistic approach to be taken by public health authorities is to assume the “worst case” scenario, with the pandemic having a high severity category, and to invoke rigorous and sustained social distancing interventions coupled with use of antiviral agents. If the severity of the pandemic is subsequently determined to be in a lower category, then interventions may be weakened by reducing the duration of social distancing measures, dropping some of the less effective and less cost effective ones, such as workplace staff reductions, and restricting the use of antivirals to a treatment-only regimen.

The results of the study hold for the given set of parameter settings, including those examined as part of the sensitivity analysis. As the model is based on the demographics, life expectancy and healthcare system of a combined rural and urban Australian community, the results should be applicable to developed countries having broadly similar population structures and healthcare systems. However differences in contact patterns, household sizes and the availability of intervention resources, for example, may prevent their applicability to low-income, developing countries, with their quite different population and healthcare characteristics. We have expressed intervention, healthcare and lost productivity costs using USA data, given that such costs in the USA and Australia are comparable. Text S1 (Table S1.11) presents overall pandemic costs and numbers of deaths and lives saved from activation of the most cost effective interventions, for the population of Australia, ∼23 million. Costs were expressed in US dollars to make the results readily transferrable to a wide range of other countries.

### Related Research

Two related studies have analysed the cost effectiveness of pandemic influenza public health mitigation strategies, using simulated pandemic outcomes coupled to a health outcomes and economic costing model [37], [61]. Although methodologically similar, this study extends upon the scope of these previous studies in the number of severity and intervention scenarios considered, and in the use of health outcome data (case fatality, hospitalization and ICU usage) observed from the 2009 pandemic. Although the two studies did not include a cost component arising from productivity loss due to death, the alternative cost effectiveness analysis presented in this paper, which similarly excludes death-related productivity losses (outlined in the Results and detailed in Text S1), allows these studies to be compared with that reported here.

In contrast to these two studies, the current study found that social distancing interventions which result in significant attack rate and (thus) mortality reduction, such as long-duration school closure, was highly cost effective for severe pandemics. However, Perlroth et al [61] found that adding continuous school closure to an antiviral strategy always resulted in increased total cost, even for pandemics with high transmissibility (R = 2.1) and a case fatality ratio up to 2%. Similarly Sander et al [37] found that the addition of continuous school closure to an extended antiviral strategy also increased total costs, including pandemics with a 5% case fatality ratio. These studies thus concluded that rigorous social distancing in the form of long-duration school closure represents a tradeoff between economic cost and effectiveness in saving lives. By contrast, we found that for a pandemic with R of 1.8, adding continuous school closure to an antiviral treatment and extended prophylaxis strategy was cost effective (i.e. it both reduced costs and saved lives) when the case fatality ratio was 1.5% or greater; that is, for severity categories 4 and 5.

The reason for this difference is due to these related studies not including higher costs which may reasonably be associated with higher pandemic severity. The approach taken in this study assumed increased hospitalisation and ICU rates occurring along with higher CFRs, whilst the related studies have hospitalisation rates fixed to the attack rate, which is independent of pandemic severity. Rates of severe health outcomes which incurred medical costs (hospitalization in Perlroth et al. [61], and pneumonia and bronchitis in Sander et al. [37]) were assumed to be proportional to the attack rate, but not proportional to the case fatality ratio, as adopted in this study. As a result, potential savings in medical costs due as a result of interventions were no higher for the more severe pandemics, and there was no pandemic severity at which the more expensive interventions, such as continuous school closure, became cost effective.

A previous paper by the authors examined the cost effectiveness of interventions used in the “mild” 2009 pandemic, which assumed a very low CFR of 0.006% and a reproduction number of 1.2 [15], derived from data supplied by the Health Department of Western Australia. Comprehensive analyses by others of the probable CFR for the 2009 pandemic [28] resulted in us defining a category 0 CFR of 0.03% to represent the severity of this pandemic.

## Conclusions

Historical evidence from the 1918/1919 pandemic [62], [63] and recent modelling research [13], [48], [49], [51], [64]–[67] have shown that rigorous and sustained social distancing is capable of effectively mitigating illness and death due to pandemic influenza. The resulting societal disruption and associated productivity losses would make such interventions unpopular, yet the results of this study give evidence as to both the potential effectiveness and cost effectiveness of using such rigorous and sustained interventions during a future, severe pandemic.

Where the severity of an emerging pandemic is initially unknown, the results indicate that assuming severity to be high is the preferred option. The most appropriate intervention in this situation is continuing school closure and community contact reduction combined with antiviral drug treatment and household prophylaxis. This combination of interventions is included in all the most cost effective strategies. When the severity of the strain has been determined, alternative strategies which trade off effectiveness for cost may then be adopted. If the severity is determined to be low, public health authorities may consider relaxing strict and sustained school closure measures, for example.

## Supporting Information

Text S1 **Additional model detail and sensitivity analysis results.**
(PDF)
