# Supplementary material for: The Cost Effectiveness of Pandemic Influenza Interventions: A Pandemic Severity Based Analysis
Source: PLoS One. 2013 Apr 9;8(4):e61504. doi: 10.1371/journal.pone.0061504 (PMC3621766; doi:10.1371/journal.pone.0061504)
Supplement: Text S1 — Additional model detail and sensitivity analysis results. (PDF) [file pone.0061504.s001.pdf]

The cost effectiveness of pandemic influenza interventions: a pandemic severity based analysis

George J Milne , Nilimesh Halder , Joel K Kelso

Supporting Information Text S1

Simulation Model

Population contact network

The simulation model captures the contact dynamics of the population of Albany, Western Australia using census, state and local government data [1], allowing the replication of the individual age and household structure of all households in this town of approximately 30,000 individuals. Human contact networks were modelled as a network of connected households and contact hubs such as schools, childcare centres, workplaces and a regional hospital. Individuals in each household and hub made contacts within a close contact mixing group, taken to be the entire household or a subset of larger hubs, and also made additional non-hub based random contacts in the wider community. Using this community-based population model, we conducted stochastic, individual-based spatial simulations of an influenza epidemic. We assumed that an average of one new infection per day was stochastically introduced into the population during the whole period of the simulations. The simulation period was divided into 12 hour day/night cycles and during each simulation cycle a nominal location for each individual was determined. This took into consideration the cycle type (day/night, weekday/weekend), infection state of each individual and whether child supervision was needed to look after a child at home. Individuals occupying the same location during the same time period (cycle) were assumed to come into potential infective contact. Details of the underlying model are presented in [2] and in that reference's online supporting material.

Influenza transmission model

In the simulation model we assumed that infectious transmission could occur when an infectious and susceptible individual came into contact during a simulation cycle. Following each contact a new infection state for the susceptible individual (either to remain susceptible or to become infected) was randomly chosen via a Bernoulli trail. Once infected an individual progressed through a series of infection states according to a fixed timeline.

The probability that a susceptible individual would be infected by an infectious individual was calculated according to the following transmission function, which takes into account the disease infectivity of the infectious individual I_i and the susceptibility of susceptible individual I_s at the time of contact.

$$P_{trans}(I_i,I_s) = \beta \times Inf(I_i) \times Susc(I_s) \times AVF(I_i,I_s)$$

The baseline transmission coefficient β was initially chosen to give an epidemic with a final attack rate of 17.4% which is consistent with seasonal influenza as estimated in Table 3 of [3]. To achieve simulations under a range of basic reproduction numbers (R_0), β was increased from this baseline value to achieve epidemics of various R_0 magnitudes; details of the procedure for estimating β and R_0 are given in [2]. A pandemic with a reproduction number of 1.5 corresponds to some estimations of the basic reproduction number of the 2009 H1N1 pandemic [4-7], while a reproduction number of 2.5 corresponds to some estimates of what may have occurred in the 1918/1919 pandemic [8,9].

The disease infectivity parameter $Inf(I_i)$ was set to 1 for symptomatic individuals at the peak period of infection and then to 0.5 for the rest of the infectivity period The infectiousness of asymptomatic individuals is also assumed to be 0.5 and this applies to all infected individuals after the latent period but before onset of symptoms. The infection profile of a *symptomatic* individual was assumed to last for 6 days as follows: a 0.5 day latent period (with $Inf(I_i)$ set to 0) is followed by 1 day asymptomatic and infectious, where $Inf(I_i)$ is set to 0.5; then 2 days at peak infectiousness (with $Inf(I_i)$ set to 1.0); followed by 2.5 days reduced infectiousness (with $Inf(I_i)$set to 0.5). For an infected but *asymptomatic* individual the whole infectious period (of 5.5 days) is at the reduced level of infectiousness with $Inf(I_i)$ set to 0.5. This infectivity profile is a simplification of the infectivity distribution found in a study of viral shedding [10]. As reported below in the results section for the unmitigated no intervention scenario, these assumptions regarding the duration of latent and infectious periods lead to a mean generation time (serial interval) of 2.47 days which is consistent with that estimated for H1N1 2009 influenza [4].

Following infection an individual is assumed to be immune to re-infection for the duration of the simulation. We further assume that influenza symptoms develop one day into the infectious period [10], with 20% of infections being asymptomatic among children and 32% being asymptomatic among adults. These percentages were derived by summing the age-specific antibody titres determined in Table 5 of [11]. Symptomatic individuals will withdraw into the home with the following probabilities; adults 50% and children 90%, which is in keeping with the work of [12,13].

The susceptibility parameter $Susc(I_s)$ is a function directly dependent on the age of the susceptible individual. It captures age-varying susceptibility to transmission due to either partial prior immunity or age-related differences in contact behaviour. To achieve a realistic age specific infection rate, the age-specific susceptibility parameters were calibrated against the serologic infection rates for seasonal H3N2 in 1977-1978 in Tecumseh, Michigan [3]. The resulting age-specific attack rates are consistent with H1N1 2009 influenza [14,15], with a higher attack rate in children and young adults (details of the calibration procedure may be found in [2]).

The antiviral efficacy factor $AVF(I_i,I_s)$ = $(1 - AVE_i)*(1 - AVE_s)$ represents the potential reduction in infectiousness of an infected individual (denoted by AVE_i) induced by antiviral treatment, and the reduction in susceptibility of a susceptible individual (denoted by AVE_s) induced by antiviral prophylaxis. When

no antiviral intervention was administrated the values of both AVE_i and AVE_s were assumed to be 0, indicating no reduction in infectiousness or susceptibility. However, when antiviral treatment was being applied to the infectious individual the value of AVE_i was set at 0.66, capturing a reduction in infectiousness by factor of 66% [16]. Similarly, when the susceptible individual was undergoing antiviral prophylaxis the value of AVE_s was set to 0.85 indicating a reduction in susceptibility by a factor of 85% [16]. This estimate is higher than most previous modelling studies, which assume an AVE_s of 30%. This common assumption appears to stem from an estimate made in [17] based on 1998-1999 trial data. Our higher value is based on a more comprehensive estimation process reported in [16], which also incorporated data from an additional study performed in 2000-2001 [18]. It is also in line with estimates of 64%-89% reported in [19].

Intervention Strategies

We examined a comprehensive range of intervention strategies including school closure, antiviral drugs for treatment and prophylaxis, workplace non-attendance (workforce reduction) and community contact reduction. These interventions were considered individually and in combination and social distancing interventions were considered for either continuous periods (that is, until the local epidemic effectively ceased) or periods of fixed duration (2, 4 or 8 weeks).

Antiviral drug interventions and social distancing interventions were initiated when specific threshold numbers of symptomatic individuals were diagnosed in the community, and this triggered health authorities to mandate the intervention response. This threshold was taken to be 0.1% of the population. It was assumed that 50% of all symptomatic individuals were diagnosed, and that this diagnosis occurred at the time symptoms appeared.

For continuous school closure, all schools were closed simultaneously once the intervention trigger threshold was reached. For fixed duration (e.g. 2 weeks or 8 weeks) school closure, schools were closed individually as follows: for a primary school the whole school was closed if 1 or more cases were detected in the school; in a high school only the class members of the affected class were isolated (sent home and isolated at home) if no more than 2 cases were diagnosed in a single class; however if there were more than 2 cases diagnosed in the entire high school the school was closed. Note that these school closure policies were only activated after the community-wide diagnosed case threshold was reached; cases occurring in schools before this time did not result in school closure. This policy of triggering school closure based on epidemic progression avoids premature school closure which can reduce the effectiveness of limited duration school closure; see [20] for a detailed description of proposed school closure strategies.

Three antiviral drug strategies have been examined; antiviral drugs used solely for treatment of symptomatic cases (strategy T), T plus prophylaxis of all household members of a symptomatic case (strategy T+H), and T+H plus prophylaxis applied to the extended contact group (such as school or workplace contacts) of a symptomatic case (strategy T+H+E). Antiviral treatment (and prophylaxis for household or work / school group contacts) was assumed to

begin 24 hours after the individual became symptomatic. It was assumed that an individual would receive at most one prophylactic course of antiviral drugs. Further details of antiviral interventions are given in [21,22].

Workforce reduction (WR) was modelled by assuming that for each day the intervention was in effect each worker had a 50% probability of staying at home and thus did not make contact with co-workers. Community contact reduction (CCR) was modelled by assuming that on days when the intervention was in effect all individuals made 50% fewer random community contacts. The most rigorous social distancing interventions considered in this study, which we denote as strict social distancing, involve the combined activation of school closure with workforce reduction and/or community contact reduction, and for this to occur for significant time periods; continuous and 8 weeks duration were considered.

Extended Prophylaxis and Rigorous Social Distancing

For strategies including rigorous social distancing (continuous school closure and community contact reduction), adding extended prophylaxis to antiviral treatment and household prophylaxis results in a slightly higher attack rate – this can be seen in Table 2 of the main paper.

This occurred because it was assumed that each individual would receive at most one prophylactic antiviral course: many prophylactic courses were used early on as a result of infections of school and work peers (which did not present a high transmission risk due to rigorous social distancing) and were then unavailable later when a household member (presenting a high transmission risk) became infected.

The result of extending prophylaxis to school and workplace contact groups in this scenario was that more infections occurred overall as prophylactic courses were used less efficiently, but that a slightly lower number of life years were saved as the infection (and mortality) burden was shifted slightly away from working adults to children. At lower severity categories this shift also resulted in a slightly lower total cost, as household and extended prophylaxis prevented additional lost work days compared to household prophylaxis.

Cost Effectiveness without Death Related Productivity Losses

The costing model used for the analysis in the main paper included future productivity losses arising from deaths caused by the pandemic. An alternative cost analysis for all interventions and severity categories omitting this cost component was also conducted. Figure S1.1 shown below is a counterpart to Figure 1 in the main paper, showing the cost effectiveness of interventions for severity categories 0, 1, 3 and 5. Table S1.1 presents the total cost and cost per life years saved for each intervention strategy and all six severity categories.

Figure S1.1 Intervention cost effectiveness plain without death-related productivity losses

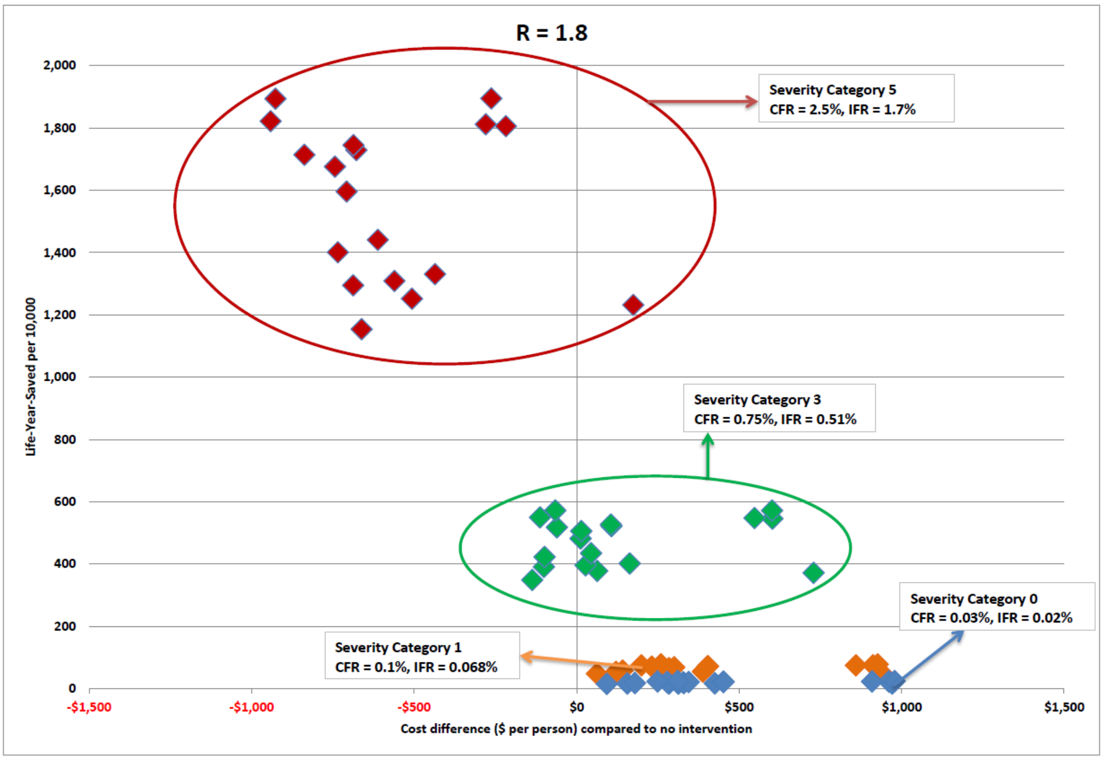

Cost effectiveness for each intervention strategy is plotted as a point in a two-dimensional plane, with points coloured by severity category: blue points - category 0 (case fatality ratio 0.03%), orange points - category 1 (case fatality ratio 0.1%), green points - category 3 (case fatality ratio 0.75%), red points - category 5 (case fatality ratio 2.5%). Horizontal axis represents the cost of the intervention strategy as a difference in total cost between two scenarios; an outbreak with the intervention in place and an outbreak with no interventions, expressed as dollars per member of the population. Vertical axis represents the number of life years saved by each strategy: the difference in life years lost for an outbreak with and without the strategy in place, expressed as life years saved per 10,000 population and also discounted.

Table S1.1 Cost effectiveness and total cost of interventions without death related productivity losses

Intervention Strategy	AR (%)	Cat 0 CFR=0.03% IFR = 0.02%	Cat 1 CFR=0.1% IFR = 0.068%	Cat 2 CFR=0.25% IFR = 0.17%	Cat 3 CFR=0.75% IFR = 0.51%	Cat 4 CFR=1.5% IFR = 1.02%	Cat 5 CFR=2.5% IFR = 1.7%
**no intervention**	**32**	$90	$151	$236	$531	$963	$1,538
**SC 2 wks + T+H+E**	**15**	**$126,405**	**$44,541**	**$21,809**	**$11,302**	**$8,663**	**$7,586**
		$184	$213	$253	$394	$600	$875
***SC cont + WR cont**	**15**	**$684,521**	**$213,672**	**$90,850**	**$33,830**	**$19,636**	**$13,890**
		$1,062	$1,089	$1,127	$1,259	$1,453	$1,711
**SC 8 + WR 4 + CCR 4 + T+H**	**14**	**$255,793**	**$82,923**	**$36,996**	**$15,702**	**$10,387**	**$8,230**
		$403	$429	$466	$594	$781	$1,030
**SC 8 wks + T+H**	**14**	**$151,070**	**$50,694**	**$23,552**	**$10,984**	**$7,839**	**$6,560**
		$246	$272	$307	$430	$610	$850
***SC cont + CCR 4**	**14**	**$227,324**	**$73,842**	**$33,024**	**$14,101**	**$9,378**	**$7,460**
		$375	$400	$435	$558	$737	$977
***SC cont + WR 4 + CCR 4**	**13**	**$307,246**	**$97,970**	**$42,821**	**$17,237**	**$10,859**	**$8,273**
		$515	$539	$574	$693	$868	$1,101
**SC 8 wks + T+H+E**	**12**	**$152,642**	**$50,310**	**$22,894**	**$10,191**	**$7,017**	**$5,727**
		$269	$292	$323	$432	$591	$803
**SC 8 + WR 4 + CCR 4 + T+H+E**	**11**	**$231,917**	**$74,131**	**$32,501**	**$13,190**	**$8,376**	**$6,424**
		$421	$442	$472	$574	$725	$925
**SC cont + T+H**	**9**	**$208,073**	**$65,936**	**$28,591**	**$11,263**	**$6,946**	**$5,196**
		$418	$435	$459	$543	$666	$829
**SC cont + T+H+E**	**8**	**$206,446**	**$64,993**	**$27,946**	**$10,752**	**$6,469**	**$4,734**
		$436	$451	$472	$545	$651	$794
***SC cont + CCR cont**	**7**	**$170,698**	**$53,911**	**$23,277**	**$9,061**	**$5,519**	**$4,084**
		$368	$382	$402	$469	$568	$700
**SC cont + WR 4 + CCR 4 + T+H+E**	**7**	**$248,586**	**$77,523**	**$32,921**	**$12,213**	**$7,059**	**$4,973**
		$541	$554	$573	$638	$733	$860
**SC cont + WR 4 + CCR 4 + T+H**	**7**	**$246,670**	**$76,866**	**$32,608**	**$12,060**	**$6,945**	**$4,876**
		$542	$555	$573	$636	$728	$851
***SC cont + WR cont + CCR cont**	**6**	**$462,411**	**$142,236**	**$59,288**	**$20,760**	**$11,179**	**$7,305**
		$1,052	$1,063	$1,079	$1,133	$1,213	$1,319
**SC cont + CCR cont + T+H+E**	**6**	**$148,102**	**$46,494**	**$19,918**	**$7,583**	**$4,511**	**$3,267**
		$340	$350	$365	$417	$494	$595
**SC cont + WR cont + CCR cont + T+H+E**	**6**	**$437,949**	**$134,733**	**$56,173**	**$19,684**	**$10,609**	**$6,940**
		$999	$1,010	$1,025	$1,078	$1,155	$1,258
**SC cont + CCR cont + T+H**	**5**	**$168,161**	**$52,303**	**$22,132**	**$8,123**	**$4,637**	**$3,226**
		$401	$410	$422	$465	$528	$611
**SC cont + WR cont + CCR cont + T+H**	**5**	**$447,832**	**$137,402**	**$57,073**	**$19,759**	**$10,481**	**$6,730**
		$1,068	$1,077	$1,089	$1,131	$1,193	$1,275

Cost per LYS per member of the population (bold) and total pandemic cost are shown for each intervention strategy and each severity category. LYS are discounted. Values are for pandemic with unmitigated transmissibility of R = 1.8. Interventions abbreviated as: SC – school closure; CCR – 50% community contact reduction; WR – 50% workforce reduction; 4, 8 – intervention duration in weeks; cont – continuous duration; T – antiviral treatment of diagnosed symptomatic cases; H – antiviral prophylaxis of household members of diagnosed symptomatic cases, E – antiviral prophylaxis of school class or workplace contacts of diagnosed symptomatic cases. Pure social distancing interventions marked by *.

Compared to the costing methodology used in the main paper, the total cost of pandemics is lower when productivity losses due to pandemic-related deaths (DRPL) are excluded. The overall cost is reduced by a factor ranging from 2.9 at category 1 to 5.5 at category 5, for unmitigated pandemics. Tables S1.2 and S1.3 present the overall outcomes (LYS, total costs with/without DRPL, cost

breakdowns, and cost per LYS with/without DRPL) for both costing methodologies and all simulated intervention strategies for severity category 1 and 5 respectively.

If DRPL costs are excluded, the strong correlation for high severity pandemics between intervention effectiveness (manifested as a reduction in deaths and an increase in life years saved) and the reduction in total pandemic cost becomes less pronounced. This can be seen by comparing the red (category 5) set of interventions in the cost effectiveness planes in Figure S1.1 with those in Figure 1 in the main paper. For categories 3 and above the *relative* cost effectiveness of strategies within a given severity category remains unchanged, and the results are thus independent of which cost analysis method is used. Hence for high severity pandemics, the inclusion or exclusion of death related productivity losses (DRPL) leaves the qualitative outcome of the analyses unchanged.

For both cost effectiveness methodologies, and for all pandemic severity categories, the most cost effective intervention strategies are the same, namely continuous school closure and community contact reduction coupled with the use of antiviral agents for treatment and prophylaxis.

For lower severity pandemics (categories 1 and 2) the relative cost effectiveness of interventions differs slightly to that in the main paper, where DRPL costs are included. Strategies which combine school closure and antiviral measures are the most cost effective. Within this class of interventions, increasing the duration of school closure (from 2 weeks to 8 weeks to continuous closure) saves an increasing number of lives while slightly increasing the total pandemic cost. This contrasts with the analyses in the main paper where DRPL were included. In this case continuous social distancing interventions were significantly more cost effective (judged in terms of cost per LYS) than those of limited duration.

Although certain interventions with different durations of social distancing have similar cost effectiveness ratios for low severity pandemics, interventions with continuous social distancing are found to be more effective at reducing the attack rate and consequential mortality rates. For example, for category 1 pandemics a strategy of 2 weeks school closure with antiviral measures has a similar cost per LYS ratio to continuous school closure, community contact reduction and antiviral measures; however the latter results in 62% more life years saved.

Table S1.2 Life-Year Saved (LYS), total pandemic costs and cost per LYS with and without death related productivity losses and cost breakdowns for all simulated interventions for severity category 1

	Category 1 (CFR 0.1%)								
Interventions	LYS per 10,000	Total Cost with DRPL	Total cost without DRPL	Cost breakdowns				Cost per LYS (with DRPL)	Cost per LYS (without DRPL)
				HCC	AVC	ISDPL	DRPL		
**no intervention**	-	**$441**	**$151**	$86	$0	$65	$290	-	-
**T**	12	**$418**	**$165**	$74	$26	$65	$253	$352,030	$138,940
***SC 2 weeks**	15	**$411**	**$170**	$72	$0	$98	$241	$269,835	$111,171
**T+H**	27	**$383**	**$177**	$60	$54	$63	$206	$143,815	$66,373
***SC 8 + CCR 4**	30	**$447**	**$251**	$58	$0	$193	$196	$151,184	$84,898
***SC 8 + WR 4 + CCR 4**	28	**$595**	**$394**	$59	$0	$335	$201	$210,049	$139,362
***SC 8 weeks**	10	**$422**	**$238**	$55	$0	$183	$184	$126,296	$71,428
***SC 8 + WR 4**	36	**$551**	**$376**	$52	$0	$324	$175	$152,305	$104,076
**T+H+E**	38	**$355**	**$186**	$50	$76	$60	$169	$92,963	$48,698
**SC 2 wks + T+H**	39	**$366**	**$200**	$49	$52	$99	$166	$93,175	$50,891
***SC Cont**	41	**$574**	**$416**	$48	$0	$368	$158	$138,487	$100,385
***SC cont + WR 4**	47	**$692**	**$550**	$43	$0	$507	$142	$148,715	$118,266
**SC 2 wks + T+H+E**	48	**$351**	**$213**	$41	$74	$97	$139	$73,650	$44,541
***SC cont + WR cont**	51	**$1,217**	**$1,089**	$39	$0	$1,050	$128	$238,774	$213,672
**SC 8 + WR 4 + CCR 4 + T+H**	51	**$555**	**$429**	$37	$50	$342	$126	$107,277	$82,923
**SC 8 wks + T+H**	54	**$392**	**$272**	$36	$50	$186	$120	$73,085	$50,694
***SC cont + CCR 4**	54	**$518**	**$400**	$36	$0	$364	$118	$95,606	$73,842
***SC cont + WR 4 + CCR 4**	55	**$654**	**$539**	$35	$0	$505	$115	$118,862	$97,970
**SC 8 wks + T+H+E**	58	**$398**	**$292**	$32	$73	$187	$106	$68,609	$50,310
**SC 8 + WR 4 + CCR 4 + T+H+E**	60	**$543**	**$442**	$30	$72	$340	$101	$91,104	$74,131
**SC cont + T+H**	66	**$516**	**$435**	$24	$48	$363	$81	$78,133	$65,936
**SC cont + T+H+E**	69	**$521**	**$451**	$21	$70	$359	$70	$75,083	$64,993
***SC cont + CCR cont**	71	**$447**	**$382**	$20	$0	$363	$65	$63,100	$53,911
**SC cont + WR 4 + CCR 4 + T+H+E**	72	**$618**	**$554**	$19	$69	$466	$63	$86,377	$77,523
**SC cont + WR 4 + CCR 4 + T+H**	72	**$616**	**$555**	$12	$22	$1,045	$41	$85,337	$76,866
***(SC + WR + CCR) cont**	75	**$1,116**	**$1,063**	$16	$0	$1,047	$53	$149,344	$142,236
**SC cont + CCR cont + T+H+E**	75	**$402**	**$350**	$15	$68	$267	$51	$53,302	$46,494
**(SC + WR + CCR) cont + T+H+E**	75	**$402**	**$1,010**	$15	$68	$267	$51	$141,754	$134,733
**SC cont + CCR cont + T+H**	78	**$402**	**$410**	$15	$68	$267	$51	$57,626	$52,303
**(SC + WR + CCR) cont + T+H**	78	**$1,119**	**$1,077**	$12	$46	$1,018	$42	$142,730	$137,402

Life-years saved (LYS) expressed as years per 10,000 members of population. Total costs with death related productivity losses (bold) and total costs without death related productivity losses (bold shaded column) are expressed as dollars per member of population. Cost breakdowns and cost per LYS with and without (shaded column) death related productivity losses are also expressed as dollars per member of population for each intervention strategy. Both costs (death related productivity losses) and LYS are discounted. Both costs (death related productivity losses) and LYS are discounted. Cost categories are abbreviated as follows: HCC – health care costs (GP visits, hospitalisation and ICU usage), AVC – antiviral costs (cost of drugs, dispensing and stockpile renewal), ISDPL – illness and social distancing productivity losses, DRPL – death-related productivity losses. Interventions abbreviated as: SC – school closure; CCR – 50% community contact reduction; WR – 50% workforce reduction; 4, 8 – intervention duration in weeks; cont – continuous duration; T – antiviral treatment of diagnosed symptomatic cases; H – antiviral prophylaxis of household members of diagnosed symptomatic cases, E – antiviral prophylaxis of school class or workplace contacts of diagnosed symptomatic cases. Pure social distancing interventions are marked by *.

Table S1.3 Life-Year Saved (LYS), total pandemic costs and cost per LYS with and without death related productivity losses and cost breakdowns for all simulated interventions for severity category 5

	Category 5 (CFR 2.5%)								
Interventions	LYS per 10,000	Total Cost with DRPL	Total cost without DRPL	Cost breakdowns				Cost per LYS (with DRPL)	Cost per LYS (without DRPL)
				HCC	AVC	ISDPL	DRPL		
**no intervention**	-	**$8,550**	**$1,538**	$1,472	$0	$65	$7,013	-	-
**T**	287	**$7,478**	**$1,365**	$1,275	$26	$65	$6,113	$260,692	$47,599
***SC 2 weeks**	368	**$7,167**	**$1,329**	$1,231	$0	$98	$5,838	$194,777	$36,114
**T+H**	644	**$6,139**	**$1,154**	$1,038	$54	$63	$4,985	$95,374	$17,930
***SC 8 + CCR 4**	715	**$5,931**	**$1,193**	$1,000	$0	$193	$4,738	$82,973	$16,688
***SC 8 + WR 4 + CCR 4**	684	**$6,191**	**$1,353**	$1,018	$0	$335	$4,838	$90,464	$19,775
***SC 8 weeks**	485	**$5,559**	**$1,131**	$948	$0	$183	$4,428	$68,881	$14,013
***SC 8 + WR 4**	875	**$5,440**	**$1,221**	$897	$0	$324	$4,219	$62,194	$13,963
**T+H+E**	924	**$5,080**	**$990**	$854	$76	$60	$4,089	$54,989	$10,723
**SC 2 wks + T+H**	949	**$5,000**	**$989**	$838	$52	$99	$4,011	$52,712	$10,428
***SC Cont**	1002	**$5,005**	**$1,188**	$820	$0	$368	$3,817	$49,959	$11,858
***SC cont + WR 4**	1126	**$5,440**	**$1,242**	$734	$0	$507	$3,427	$41,487	$11,038
**SC 2 wks + T+H+E**	1154	**$4,233**	**$875**	$704	$74	$97	$3,358	$36,695	$7,586
***SC cont + WR cont**	1232	**$4,804**	**$1,711**	$661	$0	$1,050	$3,092	$38,994	$13,890
**SC 8 + WR 4 + CCR 4 + T+H**	1252	**$4,079**	**$1,030**	$638	$50	$342	$3,048	$32,585	$8,230
**SC 8 wks + T+H**	1295	**$3,750**	**$850**	$614	$50	$186	$2,900	$28,950	$6,560
***SC cont + CCR 4**	1309	**$3,826**	**$977**	$612	$0	$364	$2,849	$29,225	$7,460
***SC cont + WR 4 + CCR 4**	1331	**$3,882**	**$1,101**	$597	$0	$505	$2,781	$29,165	$8,273
**SC 8 wks + T+H+E**	1401	**$3,367**	**$803**	$543	$73	$187	$2,565	$24,026	$5,727
**SC 8 + WR 4 + CCR 4 + T+H+E**	1441	**$3,371**	**$925**	$514	$72	$340	$2,445	$23,397	$6,424
**SC cont + T+H**	1596	**$2,775**	**$829**	$418	$48	$363	$1,946	$17,394	$5,196
**SC cont + T+H+E**	1676	**$2,485**	**$794**	$364	$70	$359	$1,691	$14,823	$4,734
***SC cont + CCR cont**	1714	**$2,275**	**$700**	$337	$0	$363	$1,575	$13,273	$4,084
**SC cont + WR 4 + CCR 4 + T+H+E**	1729	**$2,390**	**$860**	$324	$69	$466	$1,531	$13,827	$4,973
**SC cont + WR 4 + CCR 4 + T+H**	1745	**$2,329**	**$851**	$314	$47	$489	$1,478	$13,347	$4,876
***(SC + WR + CCR) cont**	1806	**$2,603**	**$1,319**	$273	$0	$1,047	$1,284	$14,413	$7,305
**SC cont + CCR cont + T+H+E**	1822	**$1,835**	**$595**	$260	$68	$267	$1,240	$10,075	$3,267
**(SC + WR + CCR) cont + T+H+E**	1812	**$2,530**	**$1,258**	$263	$68	$926	$1,272	$13,961	$6,940
**SC cont + CCR cont + T+H**	1894	**$1,619**	**$611**	$214	$46	$351	$1,008	$8,550	$3,226
**(SC + WR + CCR) cont + T+H**	1895	**$2,284**	**$1,275**	$210	$46	$1,018	$1,009	$12,058	$6,730

Life-years saved (LYS) expressed as years per 10,000 members of population. Total costs with death related productivity losses (bold) and total costs without death related productivity losses (bold shaded column) are expressed as dollars per member of population. Cost breakdowns and cost per LYS with and without (shaded column) death related productivity losses are also expressed as dollars per member of population for each intervention strategy. Both costs (death related productivity losses) and LYS are discounted. Cost categories and interventions are abbreviated as for Table S1.2.

Sensitivity Analyses

Transmissibility

The transmissibility of a future influenza pandemic will strongly influence the final attack rate (and consequent mortality rate), the effectiveness of interventions, and the total cost of the pandemic. We repeated our cost effectiveness analysis for all intervention strategies and severity categories for pandemic strains having unmitigated transmission characteristics both lower and higher than our primary assumption of a basic reproduction number R = 1.8, with low transmissibility setting having R = 1.5 and high transmissibility having R = 2.5. Figure S1.2 shows cost effectiveness planes for these alternative transmissibility settings, for all interventions that reduced the final attack rate by at least 50%, for severity categories 0, 1, 3 and 5.

Figure S1.2 Cost effectiveness planes for low and high transmissibility pandemics with death related productivity losses

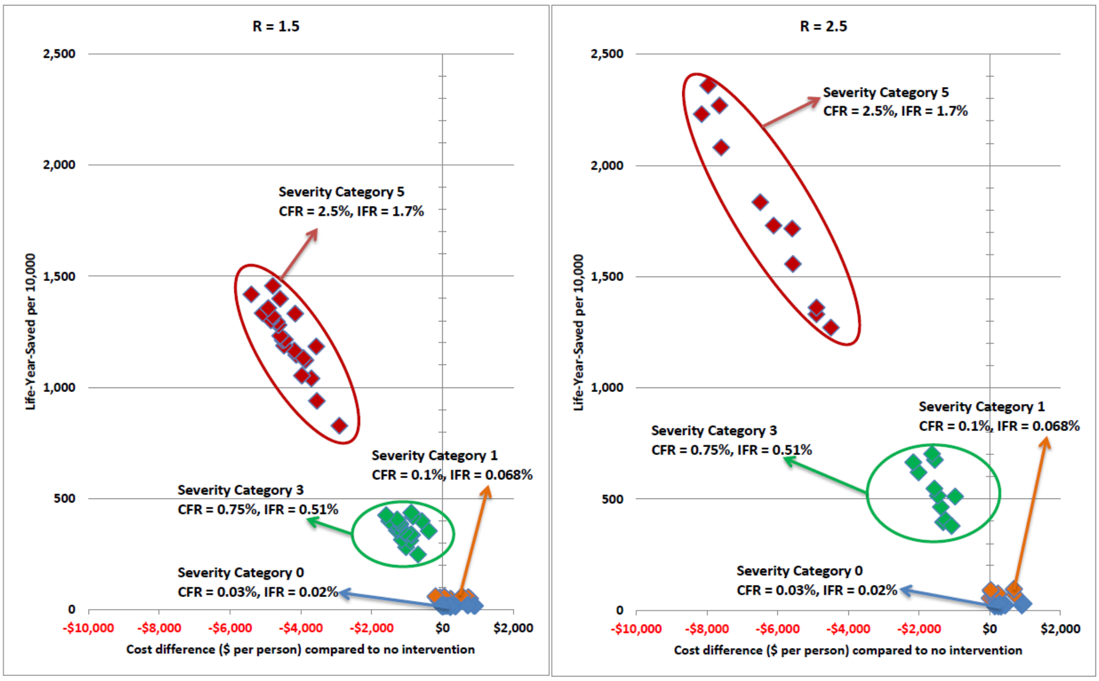

Cost effectiveness plane for alternative transmissibility assumptions: left panel, low transmissibility R = 1.5; right panel high transmissibility R = 2.5. Both costs (death related productivity losses) and LYS are discounted. All other details are as for Figure S1.1 .

Voluntary Household Isolation of Symptomatic Individuals

An underlying assumption of the simulation model is that 50% of adults (and 90% of children) who become symptomatically infected will withdraw to their home for the duration of their illness. Sensitivity analysis results for this

parameter are given in Table S1.4. Increasing self-isolation decreases infection transmission and the final attack rate (and vice versa). These results indicate that cost effectiveness (in terms of cost per life year saved) is relatively insensitive to the voluntary isolation percentage.

The largest effect occurred at high severity if 25% rather than 50% of adults self-isolated. This rendered interventions less effective and consequently less cost effective. At low severity, decreased self-isolation caused an increased in productivity losses due to workplace absenteeism, but avoids productivity losses due to illness, resulting in relatively little effect on total cost and cost effectiveness.

If 75% instead of 50% of adults self-isolated, total pandemic costs are lower, but the number of life years saved by interventions is also lower, due to the fact that the total number of life years lost lower, resulting in little change to cost effectiveness.

Intervention-Related Parameters

Sensitivity analyses were conducted to assess the impact of variation of key intervention parameters. Alternative higher and lower parameter values for antiviral efficacy, compliance to home isolation during school closure, degree of workforce reduction and degree of community contact reduction were examined and the effect which these parameter settings have on the resulting attack rate, cost and cost effectiveness of interventions were determined. Results of these analyses are presented in Tables S1.5 to S1.8 and are summarised in the main paper.

Table S1.4 Sensitivity to probability of voluntary isolation given symptoms with death related productivity losses

Voluntary isolation probability	Cat 1 (CFR – 0.1%)				
	no int.	***SC 2 wks + T + H + E***	***SC Cont + T + H + E***	***SC Cont + WR Cont + CCR Cont***	***SC Cont + CCR Cont + T + H***
**0.25 adult (0.5 child)**	39%	26%	16%	14%	9%
	$512	$490	$640	$1,214	$532
	-	$131,358	$95,559	$169,956	$61,961
Baseline **0.5 adult (0.9 child)**	32%	15%	8%	6%	5%
	$441	$351	$521	$1,116	$452
	-	$73,650	$75,083	$149,344	$57,626
**0.75 adult (1.0 child)**	27%	13%	6%	5%	5%
	$380	$320	$488	$1,099	$418
	-	$80,547	$82,108	$176,307	$67,315

Voluntary isolation probability	Cat 5 (CFR – 2.5%)				
	no int.	***SC 2 wks + T + H + E***	***SC Cont + T + H + E***	***SC Cont + WR Cont + CCR Cont***	***SC Cont + CCR Cont + T + H***
**0.25 adult (0.5 child)**	39%	26%	16%	14%	9%
	$10,233	$6,906	$4,420	$4,602	$2,645
	-	$76,638	$27,301	$26,646	$12,742
Baseline **0.5 adult (0.9 child)**	32%	15%	8%	6%	5%
	$8,550	$4,233	$2,485	$2,603	$1,619
	-	$36,695	$14,823	$14,413	$8,550
**0.75 adult (1.0 child)**	27%	13%	6%	5%	5%
	$7,158	$3,581	$1,997	$2,353	$1,692
	-	$37,322	$13,897	$15,613	$11,268

Final attack rates (%), total costs (shaded row) and cost per Life Year Saved (below shaded rows) are given for 4 intervention strategies (columns), for the baseline assumption that 50% of symptomatic adults (and 90% of symptomatic children) would stay at home, and for 2 alternate parameter values. Both costs (death related productivity losses) and LYS are discounted. Results are presented for category 1 (upper table) and category 5 (lower table). Interventions are abbreviated as for Table S1.1.

Table S1.5 Antiviral drug efficacy sensitivity analysis without death related productivity losses

	**Cat 1 (CFR – 0.1%)**												
Antiviral efficacy: infectiousness reduction (susceptibility reduction)	**no int.**	**T**		**T + H**		**T + H + E**		**SC 2 wks + T+H+E**		**SC Cont + T+H+E**		**SC Cont**	
Antiviral efficacy **11% (14%)**	**32%**		**29% (10)**		**28% (12)**		**27% (14)**		**23% (26)**		**12% (57)**		**18% (41)**
	**$151**	$26	$141	$63	$139	$93	$416	$92	$162	$87	$396	$0	$416
	-		$162,005		$168,017		$160,544		$97,082		$84,181		$100,385
Antiviral efficacy **33% (42%)**	**32%**		**28% (11)**		**26% (18)**		**23% (25)**		**20% (35)**		**10% (63)**		**18% (41)**
	**$151**	$26	$140	$61	$132	$86	$416	$85	$153	$80	$389	$0	$416
	-		$155,155		$107,711		$84,910		$67,363		$73,951		$100,385
Baseline Antiviral efficacy **66% (85%)**	**32%**		**28% (12)**		**23% (27)**		**19% (38)**		**15% (48)**		**8% (69)**		**18% (41)**
	**$151**	$26	$139	$54	$123	$76	$110	$74	$138	$70	$380	$0	$416
	-		$138,940		$66,373		$48,698		$44,541		$64,993		$100,385

	**Cat 5 (CFR – 2.5%)**												
Antiviral efficacy: infectiousness reduction (susceptibility reduction)	**no int.**	**T**		**T + H**		**T + H + E**		**SC 2 wks + T+H+E**		**SC Cont + T+H+E**		**SC Cont**	
Antiviral efficacy **11% (14%)**	**32%**		**29% (249)**		**28% (290)**		**27% (344)**		**23% (631)**		**12% (1387)**		**18% (1002)**
	**$1,538**	$26	$1366	$63	$1337	$93	$1188	$92	$1149	$87	$922	$0	$1188
	-		$55,845		$48,243		$40,525		$19,662		$7,274		$11,858
Antiviral efficacy **33% (42%)**	**32%**		**28% (260)**		**26% (434)**		**23% (600)**		**20% (853)**		**10% (1534)**		**18% (1002)**
	**$1,538**	$26	$1359	$61	$1240	$86	$1188	$85	$1002	$80	$822	$0	$1188
	-		$53,343		$29,972		$20,257		$12,745		$5,884		$11,858
Baseline Antiviral efficacy **66% (85%)**	**32%**		**28% (287)**		**23% (644)**		**19% (924)**		**15% (1154)**		**8% (1676)**		**18% (1002)**
	**$1,538**	$26	$1340	$54	$1101	$76	$914	$74	$801	$70	$723	$0	$1188
	-		$47,599		$17,930		$10,723		$7,586		$4,734		$11,858

Cost and cost effectiveness values are given for 7 intervention strategies (columns), for the baseline best-estimate antiviral efficacy values and for 2 alternative efficacy parameter settings. Final attack rates (bold face) and LYS per 10,000 member of population (in bracket) are given above shaded cells. For no intervention total cost without death related productivity losses is given below the final attack rate. For each intervention, antiviral costs are given in shaded cell followed by combined health care cost, and illness and social distancing productivity losses. Cost per LYS is given below shaded cells. LYS numbers in cost effectiveness are discounted. Results are presented for category 1 (upper table) and category 5 (lower table). Interventions are abbreviated as for Table S1.1.

Table S1.6 School closure home isolation compliance sensitivity analysis with death related productivity losses

	Cat 1 (CFR – 0.1%)						
School Closure home isolation compliance	***no int***	***SC 2 wks***	***SC Cont***	***SC Cont + T + H + E***	***SC Cont + WR Cont + CCR Cont***	***SC Cont + WR Cont + CCR Cont + T + H***	***SC Cont + CCR Cont + T + H***
Home isolation	**32%**	**27%**	**23%**	**10%**	**9%**	**5%**	**6%**
Compliance	$441	$412	$595	$511	$1,138	$1,115	$432
**50%**	-	$279,093	$214,222	$80,287	$172,347	$6,857	$57,705
Home isolation	**32%**	**27%**	**20%**	**8%**	**7%**	**5%**	**5%**
Compliance	$441	$409	$577	$511	$1,116	$1,116	$442
**75%**	-	$265,405	$154,272	$74,669	$152,540	$143,415	$57,062
Baseline Home	**32%**	**27%**	**18%**	**8%**	**6%**	**5%**	**5%**
isolation	$441	$411	$574	$521	$1,116	$1,119	$452
compliance **100%**	-	$269,835	$138,487	$75,083	$149,344	$142,730	$57,626

	Cat 5 (CFR – 2.5%)						
School Closure home isolation compliance	***no int***	***SC 2 wks***	***SC Cont***	***SC Cont + T + H + E***	***SC Cont + WR Cont + CCR Cont***	***SC Cont + WR Cont + CCR Cont + T + H***	***SC Cont + CCR Cont + T + H***
Home	**32%**	**27%**	**23%**	**10%**	**9%**	**5%**	**6%**
isolation	$8,550	$7,208	$6,240	$2,985	$3,397	$2,483	$1,910
Compliance **50%**	-	$201,827	$92,953	$19,418	$21,291	$13,497	$10,552
Home	**32%**	**27%**	**20%**	**8%**	**7%**	**5%**	**5%**
isolation	$8,550	$7,148	$5,368	$2,561	$2,740	$2,331	$1,693
Compliance **75%**	-	$191,771	$59,413	$15,494	$15,489	$12,393	$9,050
Baseline Home	**32%**	**27%**	**18%**	**8%**	**6%**	**5%**	**5%**
isolation	$8,550	$7,167	$5,005	$2,485	$2,603	$2,284	$1,619
compliance **100%**	-	$194,777	$49,959	$14,823	$14,413	$12,058	$8,550

Final attack rates (bold face, above shaded rows), total costs (shaded rows) and cost per Life Year Saved (below shaded rows) are given for 7 intervention strategies (columns), for the baseline assumption that 100% of children affected by school closure would comply with household isolation, and for 2 alternate parameter values. Both costs (death related productivity losses) and LYS are discounted. Results are presented for category 1 (upper table) and category 5 (lower table). Interventions are abbreviated as for Table S1.1 .

Table S1.7 Workforce reduction sensitivity analysis with death related productivity losses

	Cat 1 (CFR – 0.1%)			
Workforce Reduction	***no int***	***SC Cont + WR Cont***	***SC Cont + WR Cont + CCR Cont***	***SC Cont + WR Cont + CCR Cont + T+H+E***
Workforce Reduction **25%**	**32%**	**16%**	**7%**	**6%**
	$441	$887	$778	$735
	-	$187,990	$107,493	$97,479
Baseline Workforce Reduction **50%**	**32%**	**15%**	**6%**	**6%**
	$441	$1,217	$1,116	$1,063
	-	$238,774	$149,344	$141,754
Workforce Reduction **75%**	**32%**	**13%**	**5.5%**	**5%**
	$441	$1,153	$1,465	$1,423
	-	$281,753	$192,579	$186,923

	Cat 5 (CFR – 2.5%)			
Workforce Reduction	***no int***	***SC Cont + WR Cont***	***SC Cont + WR Cont + CCR Cont***	***SC Cont + WR Cont + CCR Cont + T+H+E***
Workforce Reduction **25%**	**32%**	**16%**	**7%**	**6%**
	$8,550	$4,810	$2,471	$2,162
	-	$42,167	$14,121	$11,857
Baseline Workforce Reduction **50%**	**32%**	**15%**	**6%**	**6%**
	$8,550	$4,804	$2,603	$2,530
	-	$38,994	$14,413	$13,961
Workforce Reduction **75%**	**32%**	**13%**	**5.5%**	**5%**
	$8,550	$4,772	$2,831	$2,786
	-	$35,809	$15,391	$15,133

Final attack rates (bold face, above shaded rows), total costs (shaded rows) and cost per Life Year Saved (below shaded rows) are given for 4 intervention strategies (columns), for the baseline assumption that 50% of workers would stay at home while workforce reduction was in effect, and for 2 alternate parameter values. Both costs (death related productivity losses) and LYS are discounted. Results are presented for category 1 (upper table) and category 5 (lower table). Interventions are abbreviated as for Table S1.1 .

Table S1.8 Community contact reduction sensitivity analysis with death related productivity losses

	Cat 1 (CFR – 0.1%)				
Community Contact Reduction (CCR)	***no int***	***SC Cont + CCR Cont***	***SC Cont + WR Cont + CCR Cont***	***SC Cont + WR Cont + CCR Cont + T+H+E***	***SC Cont + CCR Cont + T + H***
CCR **75%**	**32%**	**5%**	**4%**	**5%**	**5%**
	$441	$416	$1,094	$975	$367
	-	$53,520	$138,139	$125,340	$47,699
Baseline CCR **50%**	**32%**	**7%**	**6%**	**6%**	**5%**
	$441	$447	$1,116	$1,063	$452
	-	$63,100	$149,344	$141,754	$57,626
CCR **25%**	**32%**	**12%**	**10%**	**5%**	**6%**
	$441	$507	$1,157	$1,158	$478
	-	$88,246	$177,880	$149,849	$64,300

	Cat 5 (CFR – 2.5%)				
Community Contact Reduction (CCR)	***no int***	***SC Cont + CCR Cont***	***SC Cont + WR Cont + CCR Cont***	***SC Cont + WR Cont + CCR Cont + T+H+E***	***SC Cont + CCR Cont + T + H***
CCR **75%**	**32%**	**5%**	**4%**	**5%**	**5%**
	$8,550	$1,635	$2,185	$2,193	$1,666
	-	$8,694	$11,409	$11,663	$8,961
Baseline CCR **50%**	**32%**	**7%**	**6%**	**6%**	**5%**
	$8,550	$2,275	$2,603	$2,530	$1,619
	-	$13,273	$14,413	$13,961	$8,550
CCR **25%**	**32%**	**12%**	**10%**	**5%**	**6%**
	$8,550	$3,527	$3,500	$2,418	$2,001
	-	$25,419	$22,264	$12,943	$11,164

Final attack rates (bold face, above shaded rows), total costs (shaded rows) and cost per Life Year Saved (below shaded rows) are given for 4 intervention strategies (columns), for the baseline assumption that individuals would make 50% fewer community contacts while community contact reduction was in effect, and for 2 alternate parameter values. Both costs (death related productivity losses) and LYS are discounted. Results are presented for category 1 (upper table) and category 5 (lower table). Interventions are abbreviated as for Table S1.1 .

Table S1.9 Age specific susceptibility sensitivity analysis

R0 = 1.8								
	2009 H1N1 Age Specific Susceptibility				Equal Age Specific Susceptibility			
Pandemic Severity Categories	Attack Rate (%)	Life Years Saved per 10,000	Total Cost ($) per person	Cost ($) per LYS	Attack Rate (%)	Life Years Saved per 10000	Total Cost ($) per person	Cost ($) per LYS
No Intervention								
Cat 0	32	-	$179	-	32.3	-	$179	-
Cat 1	32	-	$441	-	32.3	-	$426	-
Cat 2	32	-	$943	-	32.3	-	$896	-
Cat 3	32	-	$2,649	-	32.3	-	$2,497	-
Cat 4	32	-	$5,175	-	32.3	-	$4,867	-
Cat 5	32	-	$8,550	-	32.3	-	$8,032	-
SC 8 wks + T+H								
Cat 0	14	16	$283	$173,457	10	18	$145	$79,852
Cat 1	14	54	$392	$73,085	10	59	$220	$37,004
Cat 2	14	130	$599	$45,942	10	145	$364	$25,150
Cat 3	14	391	$1,306	$33,374	10	435	$855	$19,669
Cat 4	14	778	$2,352	$30,229	10	864	$1,581	$18,294
Cat 5	14	1295	$3,750	$28,950	10	1438	$2,550	$17,732
SC cont + T+H								
Cat 0	9	20	$442	$220,269	8.3	19	$365	$188,552
Cat 1	9	66	$516	$78,133	8.3	64	$429	$67,480
Cat 2	9	161	$656	$40,789	8.3	155	$551	$35,599
Cat 3	9	482	$1,131	$23,461	8.3	464	$966	$20,817
Cat 4	9	959	$1,835	$19,143	8.3	922	$1,580	$17,132
Cat 5	9	1596	$2,775	$17,394	8.3	1536	$2,401	$15,639
SC cont + CCR cont								
Cat 0	7	21	$388	$179,889	7.5	20	$323	$162,085
Cat 1	7	71	$447	$63,100	7.5	65	$381	$58,264
Cat 2	7	173	$560	$32,466	7.5	159	$493	$30,930
Cat 3	7	518	$945	$18,250	7.5	478	$872	$18,249
Cat 4	7	1029	$1,514	$14,708	7.5	950	$1,434	$15,088
Cat 5	7	1714	$2,275	$13,273	7.5	1582	$2,184	$13,807
SC cont + CCR cont + T+H								
Cat 0	5	24	$414	$173,483	3.5	23	$334	$144,278
Cat 1	5	78	$452	$57,626	3.5	76	$362	$47,569
Cat 2	5	191	$524	$27,455	3.5	185	$415	$22,404
Cat 3	5	572	$770	$13,447	3.5	556	$596	$10,719
Cat 4	5	1138	$1,133	$9,961	3.5	1105	$863	$7,811
Cat 5	5	1894	$1,619	$8,550	3.5	1839	$1,220	$6,635

Final attack rates, life years saved (LYS) per 10,000 population, total costs per person and cost per LYS are given for 5 intervention strategies (row blocks). Results are given assuming that susceptibility to infection is uniform for all ages (grey columns), and for a 2009-like age-specific susceptibility profile (unshaded columns). Results are presented for 6 severity categories (rows). Both costs (death related productivity losses) and LYS are discounted. Interventions are abbreviated as for Table S1.1 .

Table S1.10 Asymptomatic rate sensitivity analysis

	Attack Rate, % (Infection Rate, %)		Cat 0 (CFR=0.03%)		Cat 4 (CFR=1.5%)	
Intervention Strategy	Low Asymp. Rate[1]	High Asymp. Rate[2]	Low Asymp. Rate	High Asymp. Rate	Low Asymp. Rate	High Asymp. Rate
			IFR=0.02%	IFR=0.01%	IFR=1.02%	IFR=0.6%
no intervention	32 (43)	19 (48)	-	-	-	-
			-	-	-	-
SC 2 wks + T+H+E	15 (20)	13 (33)	$155,516	$456,316	$37,772	$97,620
			15	5	693	231
*SC cont + WR cont	15 (19)	12 (29)	$709,622	$1,848,272	$44,739	$92,981
			16	7	740	315
SC 8 + WR 4 + CCR 4 + T+H	14 (18)	13 (32)	$280,148	$810,459	$34,742	$93,257
			16	5	752	255
SC 8 wks + T+H	14 (18)	12 (29)	$173,457	$419,897	$30,229	$63,377
			16	7	778	314
*SC cont + CCR 4	14 (18)	10 (25)	$249,088	$565,384	$31,143	$51,279
			16	8	786	376
*SC cont + WR 4 + CCR 4	13 (17)	10 (24)	$328,138	$712,658	$31,751	$51,496
			17	8	800	391
SC 8 wks + T+H+E	12 (16)	11 (27)	$170,942	$423,753	$25,315	$57,721
			18	7	842	335
SC 8 + WR 4 + CCR 4 + T+H+E	11 (15)	11 (29)	$248,891	$731,155	$25,349	$75,616
			18	6	866	296
SC cont + T+H	9 (12)	10 (25)	$220,269	$645,359	$19,143	$55,022
			20	8	959	367
SC cont + T+H+E	8 (10)	9 (22)	$216,534	$565,465	$16,558	$41,473
			21	9	1007	431
*SC cont + CCR cont	7 (10)	7 (18)	$179,889	$429,454	$14,708	$31,226
			21	10	1029	485
SC cont + WR 4 + CCR 4 + T+H+E	7 (9)	7 (17)	$257,440	$601,434	$15,914	$32,199
			22	11	1039	507
SC cont + WR 4 + CCR 4 + T+H	7 (9)	7 (17)	$255,140	$603,066	$15,417	$33,948
			22	10	1048	492
*SC cont + WR cont + CCR cont	6 (8)	6 (15)	$469,517	$1,073,292	$18,288	$39,165
			23	11	1085	534
SC cont + CCR cont + T+H+E	6 (7)	5 (12)	$154,908	$399,208	$11,319	$20,314
			23	12	1094	585
SC cont+WR cont+CCR cont+ T+H+E	6 (7)	4 (10)	$444,971	$1,000,741	$17,630	$31,697
			23	13	1089	600
SC cont + CCR cont + T+H	5 (6)	5 (13)	$173,483	$403,042	$9,961	$22,532
			24	12	1138	561
SC cont + WR cont + CCR cont + T+H	5 (6)	4 (11)	$453,160	$1,002,841	$15,809	$32,489
			24	12	1138	591

[1]Low Asymp. Rate – probability of asymptomatic infection average 0.3 (0.32 for adults, 0.2 for children), [2]High Asymp. Rate – probability of asymptomatic infection 0.6 (for all ages), CFR – Case Fatality rate and IFR – Infection Fatality Rate.

Final symptomatic attack rates, infection rates (in parentheses), life years saved (LYS) per 10,000 population, and cost per LYS are given for 5 intervention strategies (row blocks) are given for 19 intervention strategies (rows). Results are given assuming that 60% of infected individuals experience asymptomatic infection (shaded columns) and assuming that 30% experience asymptomatic infection (unshaded columns). Results are presented for severity categories 0 and 4. Both costs (death related productivity losses) and LYS are discounted. Interventions are abbreviated as for Table S1.1 .

Results Scaled for Australian Population

Table S1.11 Pandemic costs (with death related productivity losses) and lives saved for Australia, population ~23 million

Pandemic severity category	Most Cost Effective Strategy	Lives lost	Lives lost for no intervention	Lives saved	Total cost (M$)	Unmitigated cost (M$)	Cost difference (M$)
**Category 1**	**SC cont + CCR cont + T+H+E**	1,361	7,711	6,350	$9,238	$10,145	-$907
					$8,058	$3,472	$4,586
**Category 3**	**SC cont + CCR cont + T+H**	8,176	56,327	48,151	$17,700	$60,938	-$43,238
					$10,693	$12,203	-1,510
**Category 5**	**SC cont + CCR cont + T+H**	27,057	186,416	159,359	$37,246	$196,653	-$159,407
					$14,054	$35,364	-$21,310

The table shows the lives saved by the most cost effective strategies for three different pandemic severity categories for Australia (population ~23 million). Pandemic costs are shown with and without (shaded rows) death related productivity losses. Death related productivity losses are discounted. Interventions are abbreviated as for Table S1.1 .

Table S1.12 Intervention strategy, cost effectiveness (with death related productivity losses) and life years saved

Intervention Strategy	AR (%)	Cat 0 CFR=0.03% IFR = 0.02%	Cat 1 CFR=0.1% IFR = 0.068%	Cat 2 CFR=0.25% IFR = 0.17%	Cat 3 CFR=0.75% IFR = 0.51%	Cat 4 CFR=1.5% IFR = 1.02%	Cat 5 CFR=2.5% IFR = 1.7%
**no intervention**	**32**	-	-	-	-	-	-
		-	-	-	-	-	-
**T**	**28**	**$524,283**	**$352,030**	**$295,773**	**$270,035**	**$263,439**	**$260,692**
		4	12	29	87	172	287
***SC 2 weeks**	**27**	**$414,508**	**$269,835**	**$223,609**	**$202,438**	**$197,025**	**$194,777**
		5	15	37	111	221	368
**T+H**	**23**	**$242,799**	**$143,815**	**$113,997**	**$100,288**	**$96,809**	**$95,374**
		8	27	65	194	387	644
***SC 8 + CCR 4**	**22**	**$299,479**	**$151,184**	**$109,218**	**$89,846**	**$84,969**	**$82,973**
		9	30	72	216	429	715
***SC 8 + WP 4 + CCR 4**	**22**	**$479,944**	**$210,049**	**$136,501**	**$102,462**	**$93,936**	**$90,464**
		9	28	69	207	411	684
***SC 8 weeks**	**21**	**$251,161**	**$126,296**	**$90,973**	**$74,666**	**$70,562**	**$68,881**
		21	10	33	81	244	485
***SC 8 + WP 4**	**20**	**$356,653**	**$152,305**	**$96,888**	**$71,230**	**$64,808**	**$62,194**
		11	36	88	264	525	875
**T+H+E**	**19**	**$174,026**	**$92,963**	**$69,597**	**$58,824**	**$56,105**	**$54,989**
		12	38	93	279	555	924
**SC 2 wks + T+H**	**18**	**$180,594**	**$93,175**	**$68,279**	**$56,792**	**$53,898**	**$52,712**
		12	39	96	287	570	949
***SC Cont**	**18**	**$341,181**	**$138,487**	**$84,047**	**$58,826**	**$52,522**	**$49,959**
		13	41	101	303	602	1002
***SC cont + WP 4**	**16**	**$397,698**	**$148,715**	**$82,785**	**$52,209**	**$44,581**	**$41,487**
		14	47	113	340	676	1126
**SC 2 wks + T+H+E**	**15**	**$155,516**	**$73,650**	**$50,917**	**$40,411**	**$37,772**	**$36,695**
		15	48	116	349	693	1154
***SC cont + WR cont**	**15**	**$709,622**	**$238,774**	**$115,953**	**$58,933**	**$44,739**	**$38,994**
		16	51	124	372	740	1232
**SC 8 + WR 4 + CCR 4 + T+H**	**14**	**$280,148**	**$107,277**	**$61,350**	**$40,056**	**$34,742**	**$32,585**
		16	52	126	378	752	1252
**SC 8 wks + T+H**	**14**	**$173,457**	**$73,085**	**$45,942**	**$33,374**	**$30,229**	**$28,950**
		16	54	130	391	778	1295
***SC cont + CCR 4**	**14**	**$249,088**	**$95,606**	**$54,789**	**$35,866**	**$31,143**	**$29,225**
		16	54	132	396	786	1309
***SC cont + WR 4 + CCR 4**	**13**	**$328,138**	**$118,862**	**$63,713**	**$38,129**	**$31,751**	**$29,165**
		17	55	134	402	800	1331
**SC 8 wks + T+H+E**	**12**	**$170,942**	**$68,609**	**$41,193**	**$28,490**	**$25,315**	**$24,026**
		18	58	141	423	842	1401
**SC 8 + WR 4 + CCR 4 + T+H+E**	**11**	**$248,891**	**$91,104**	**$49,475**	**$30,164**	**$25,349**	**$23,397**
		18	60	145	435	866	1441
**SC cont + T+H**	**9**	**$220,269**	**$78,133**	**$40,789**	**$23,461**	**$19,143**	**$17,394**
		20	66	161	482	959	1596
**SC cont + T+H+E**	**8**	**$216,534**	**$75,083**	**$38,035**	**$20,840**	**$16,558**	**$14,823**
		21	69	169	506	1007	1676
***SC cont + CCR cont**	**7**	**$179,889**	**$63,100**	**$32,466**	**$18,250**	**$14,708**	**$13,273**
		21	71	173	518	1029	1714
**SC cont + WR 4 + CCR 4 + T+H+E**	**7**	**$257,440**	**$86,377**	**$41,775**	**$21,068**	**$15,914**	**$13,827**
		22	72	174	522	1039	1729
**SC cont + WR 4 + CCR 4 + T+H**	**7**	**$255,140**	**$85,337**	**$41,080**	**$20,532**	**$15,417**	**$13,347**
		22	72	176	527	1048	1745
***SC cont + WR cont + CCR cont**	**6**	**$469,517**	**$149,344**	**$66,397**	**$27,869**	**$18,288**	**$14,413**
		23	75	182	546	1085	1806
**SC cont + CCR cont + T+H+E**	**6**	**$154,908**	**$53,302**	**$26,726**	**$14,391**	**$11,319**	**$10,075**
		23	75	183	550	1094	1822
**SC cont + WR cont + CCR cont + T+H+E**	**6**	**$444,971**	**$141,754**	**$63,194**	**$26,704**	**$17,630**	**$13,961**
		23	75	183	548	1089	1812
**SC cont + CCR cont + T+H**	**5**	**$173,483**	**$57,626**	**$27,455**	**$13,447**	**$9,961**	**$8,550**
		24	78	191	572	1138	1894
**SC cont + WR cont + CCR cont + T+H**	**5**	**$453,160**	**$142,730**	**$62,401**	**$25,087**	**$15,809**	**$12,058**
		24	78	191	572	1138	1895

Final symptomatic attack rate (AR), cost per LYS (bold) and life-years saved (LYS) are shown for each intervention strategy and each severity category. LYS expressed as years per 10,000 members of population. Cost effectiveness includes death related productivity losses. Both costs (death related productivity losses) and LYS are discounted. Values are for pandemic with unmitigated transmissibility of R_0 = 1.8. Interventions abbreviated as: SC – school closure; CCR – 50% community contact reduction; WR – 50% workforce reduction; 4, 8 – intervention duration in weeks; cont – continuous duration; T – antiviral treatment of diagnosed symptomatic cases; H – antiviral prophylaxis of household members of diagnosed symptomatic cases, E – antiviral prophylaxis of school class or workplace contacts of diagnosed symptomatic cases. Pure social distancing interventions marked by *.

References

1. Australian Bureau of Statistics (2004) The 2004 Australian Census.
2. Milne GJ, Kelso JK, Kelly HA, Huband ST, McVernon J (2008) A small community model for the transmission of infectious diseases: comparison of school closure as an intervention in individual-based models of an influenza pandemic. PLoS ONE 3: e4005.
3. Monto A, Koopman J, Longini IMJ (1985) Tecumseh study of illness XIII. Influenza infection and disease, 1976-1981. American Journal of Epidemiology 121: 811-822.
4. Fraser C, Christl DA, Cauchemez S, Hanage WP, Van Kerkhove MD, et al. (2009) Pandemic potential of a strain of influenza A (H1N1): early findings. Science 324: 1557-1561.
5. Kelly H, Mercer G, Fielding J, Dowse G, Glass K, et al. (2010) Pandemic (H1N1) 2009 influenza community transmission was established in one Australian state when the virus was first identified in North America. PLoS ONE 5: e11341.
6. Nishiura H, Wilson N, Baker M (2009) Estimating the reproduction number of the novel influenza A virus (H1N1) in a Southern Hemisphere setting: preliminary estimate in New Zealand. Journal of the New Zealand Medical Association 122: 73-77.
7. Munayco C, Gomez J, Laguna-Torres V, Arrasco J, Kochel T, et al. (2009) Epidemiological and transmissibility analysis of influenza A (H1N1) v in a southern hemisphere setting: Peru. Eurosurveillance 14: pii=19299.
8. Frost W (1920) Statistics of influenza morbidity with special reference to certain factors in case incidence and case fatality. Public Heath Report 35: 584-597.
9. Glezen WP (1996) Emerging infections: pandemic influenza. Epidemiologic Reviews 18: 64-76.
10. Carrat F, Vergu E, Ferguson NM, Lemaitre M, Cauchemez S, et al. (2008) Time lines of infection and disease in human influenza: a review of volunteer challenge studies. American Journal of Epidemiology 167: 775-785.
11. Fox JP, Hall CE, Cooney MK, Foy HM (1982) Influenzavirus infections in Seattle families, 1975-1979. American Journal of Epidemiology 116: 212-227.
12. Longini IM, Jr., Nizam A, Xu S, Ungchusak K, Hanshaoworakul W, et al. (2005) Containing pandemic influenza at the source. Science 309: 1083-1088.
13. Ferguson NM, Cummings DAT, Fraser C, Cajka JC, Cooley PC, et al. (2006) Strategies for mitigating an influenza pandemic. Nature 442: 448-452.
14. Yasuda H, Suzuki K (2009) Measures against transmission of pandemic H1N1 influenza in Japan in 2009: simulation model. Eurosurveillance 14: ppi=19385.
15. ECDC (2009) ECDC Risk Assessment 2009 influenza A(H1N1) pandemic Version 7. Stockholm: European Center for Disease Control.
16. Yang Y, Longini IM, Jr., Halloran ME (2006) Design and evaluation of prophylactic interventions using infectious disease incidence data from close contact groups. Journal of Applied Statistics 55: 317-330.

17. Longini I, Halloran M, Nizam A, Yang Y (2004) Containing pandemic influenza with antiviral agents. American Journal of Epidemiology 159: 623-633.
18. Hayden FG, Belshe R, Villanueva C, Lanno R, Hughes C, et al. (2004) Management of influenza in households: a prospective, randomized comparison of oseltamivir treatment with or without postexposure prophylaxis. The Journal of Infectious Diseases 189: 440-449.
19. Moscona A (2005) Neuraminidase inhibitors for influenza. New England Journal of Medicine 353: 1363-1373.
20. Halder N, Kelso J, Milne G (2010) Developing guidelines for school closure interventions to be used during a future influenza pandemic. BMC Infectious Diseases 10: 221.
21. Halder N, Kelso J, Milne G (2010) Analysis of the effectiveness of interventions used during the 2009 H1N1 influenza pandemic. BMC Public Health 10: 168.
22. Kelso JK, Halder N, Milne GJ (2010) The impact of case diagnosis coverage and diagnosis delays on the effectiveness of antiviral strategies in mitigating pandemic influenza A/H1N1 2009. PLoS ONE 5: e13797.
